# X-box binding protein 1 as a key modulator in “healing endothelial cells”, a novel EC phenotype promoting angiogenesis after MCAO

**DOI:** 10.1186/s11658-022-00399-5

**Published:** 2022-11-08

**Authors:** Zhuohui Chen, Xiang Wang, Haiyue Wu, Yishu Fan, Zhouyi Yan, Chenxiao Lu, Hongfei Ouyang, Shiyu Zhang, Mengqi Zhang

**Affiliations:** 1 Department of Neurology, Xiangya Hospital, Central South University, 87 Xiangya Road, Changsha, 410008 China; 2 Xiangya School of Medicine, Central South University, Changsha, 410013 China; 3 National Clinical Research Center for Geriatric Disorders, Xiangya Hospital, Central South University, Changsha, 410008 Hunan China; 4 Department of Neurosurgery, Xiangya Hospital, Central South University, 87 Xiangya Road, Changsha, 410008 China

**Keywords:** Single-cell RNA sequencing, Endothelial cell, Middle cerebral artery occlusion, X-box binding protein 1, Synchrotron radiation

## Abstract

**Background:**

Endothelial cells (ECs) play an important role in angiogenesis and vascular reconstruction in the pathophysiology of ischemic stroke. Previous investigations have provided a profound cerebral vascular atlas under physiological conditions, but have failed to identify new disease-related cell subtypes. We aimed to identify new EC subtypes and determine the key modulator genes.

**Methods:**

Two datasets GSE174574 and GSE137482 were included in the study. Seurat was utilized as the standard quality-control pipeline. UCell was used to calculate single-cell scores to validate cellular identity. Monocle3 and CytoTRACE were utilized in aid of pseudo-time differentiation analysis. CellChat was utilized to infer the intercellular communication pathways. The angiogenesis ability of ECs was validated by MTS, Transwell, tube formation, flow cytometry, and immunofluorescence assays in vitro and in vivo. A synchrotron radiation-based propagation contrast imaging was introduced to comprehensively portray cerebral vasculature.

**Results:**

We successfully identified a novel subtype of EC named “healing EC” that highly expressed pan-EC marker and pro-angiogenic genes but lowly expressed all the arteriovenous markers identified in the vascular single-cell atlas. Further analyses showed its high stemness to differentiate into other EC subtypes and potential to modulate inflammation and angiogenesis via excretion of signal molecules. We therefore identified X-box binding protein 1 (*Xbp1*) as a key modulator in the healing EC phenotype. In vitro and in vivo experiments confirmed its pro-angiogenic roles under both physiological and pathological conditions. Synchrotron radiation-based propagation contrast imaging further proved that Xbp1 could promote angiogenesis and recover normal vasculature conformation, especially in the corpus striatum and prefrontal cortex under middle cerebral artery occlusion (MCAO) condition.

**Conclusions:**

Our study identified a novel disease-related EC subtype that showed high stemness to differentiate into other EC subtypes. The predicted molecule Xbp1 was thus confirmed as a key modulator that can promote angiogenesis and recover normal vasculature conformation.

**Supplementary Information:**

The online version contains supplementary material available at 10.1186/s11658-022-00399-5.

## Background

An intertwined and highly connected cerebrovascular network ensures blood flow circulation into the brain, tasked with the delivery of sufficient oxygen and glucose and removal of byproducts produced during cerebral metabolism [1]. The disconnection and loss of integrity of microvasculature is one of the main pathologies after the occurrence of ischemic stroke [2, 3], which is a leading cause of death and disability in the world [4]. Several investigations have shown that the retention of the cerebrovascular network and promotion of cerebrovascular reconstruction help mitigate the injury and restore the neuronal function [5–7]. Therein, angiogenesis serves as one of the most important repair mechanisms.

Endothelial cells (ECs) serve as the workhorse in angiogenesis [8, 9]. The external pressure induced by diseases like stroke influences the angiogenesis abilities of ECs [10]. However, like other vascular beds, diverse cell populations and distinct functions also exist in the cerebral endothelial cells along the contiguous segments [11–13]. The cerebral vessels are also composed of various other cells, including smooth muscle cells (SMCs), pericytes, and perivascular fibroblast cells. Moreover, the brain microenvironment formed by glial cells, neurons, and infiltrating peripheral immune cells interacts closely with ECs [1, 14, 15]. Therefore, identifying the EC subpopulations and their interactions with other cells in brain microenvironment may lay the groundwork for research into the pathophysiology of ischemic stroke and development of drug targets.

Single-cell RNA sequencing (scRNA-seq) has rapidly become a powerful tool for the exploration of various cell types, including ECs, in mouse brain and has generated a detailed mouse brain and vascular cell atlas [12, 13, 16]. However, most of these data were generated under physiological conditions, and thus overlooked the disease-related cell subtypes or phenotypes. Recent studies exploring neurologic diseases such as arteriovenous malformation [17, 18] and Alzheimer’s disease [19] have indicated that cellular phenotypes and functions could be altered during pathological events. In the context of ischemic stroke, a previous investigation interpreted the data on the basis of the physiological atlas and failed to identify new disease-related cell subtypes [20]. To fill this gap, we reanalyzed the data generated from a mouse middle cerebral artery occlusion (MCAO) model, and focused on identifying new disease-related EC subtypes or phenotypes under ischemic stroke condition. We successfully discovered a new EC subtype that highly expressed pan-EC marker genes and pro-angiogenic factors but underexpressed all the arteriovenous markers identified in the vascular cell atlas. This EC subtype also possessed high differentiation potency into known ECs. We thus named this new EC subtype “healing EC.” We further identified a key molecule *Xbp1* that highly correlated with the phenotype of healing ECs. A series of in vitro and in vivo analyses confirmed the roles of *Xbp1* in promoting angiogenesis. In addition, synchrotron radiation-based propagation contrast imaging (SR-PCI) was utilized and enabled us to meticulously assess the details of the microvascular network at whole-brain and regional scales under various conditions. Vessels with a minimal diameter of ~ 10 μm can thus be discriminated in rodents [21]. Our findings gave us insight into the roles of Xbp1 in modulating healing ECs and promoting angiogenesis after MCAO.

## Methods

### In silico analyses

#### Data access and acquisition

The 10x Genomics single-cell RNA sequencing (scRNA-seq) data and bulk RNA sequencing data were acquired through the Gene Expression Omnibus (GEO) database (https://www.ncbi.nlm.nih.gov/geo/, accession numbers GSE174574 and GSE137482) [20, 22]. The scRNA-seq dataset was generated using brain tissues of C57BL/6 mice aged 6–8 weeks under post-MCAO (24 h, *n* = 3) and sham (*n* = 3) conditions. The preprocessed 10x spare matrix (barcodes, genes, and matrix) was utilized in the current study. The bulk dataset was generated using brain tissues of C57BL/6 mice aged 3 months and 18 months under post-MCAO (3 days) and control conditions. Each group yielded six replicates. The normalized count matrix was utilized in the current study.

#### Quality control of scRNA-seq data

Seurat version 4.1.0 was used to create a Seurat object for further analyses in R-dev version [23]. The raw scRNA-seq matrix included 58,528 cells and 19,290 genes in total, with a mean read depth of 92,207 reads per cell, a median of 2610 unique molecular identifier count per cell, and a median of 1295 genes per cell. For quality control (QC), the following filter standards were set: (1) cells were excluded with detected genes (nFeature) < 300, percentage of mitochondrial gene expression > 15%, percentage of ribosomal gene expression < 3%, and percentage of hemoglobin gene expression > 0.1%; (2) genes with expression in fewer than three cells were excluded, and housekeeping gene *Malat1* and mitochondrial genes were excluded to avoid interference; (3) for each sample, DoubletFinder version 2.0.3 with recommended parameter and an expectation of 4% doublets was used to exclude the predicted doublets from further analyses [24]. After QC, a total of 52,809 cells and 19,276 genes were included for further analyses.

#### Data integration, dimensional reduction, visualization, and cell type annotation

The integrated dataset was log-normalized and scaled using Seurat workflow [23]. Harmony version 0.1.0 was used to integrate the scRNA-seq of the six samples and reduce the batch effects caused by sequencing batch [25]. Principal component analysis (PCA), *t*-distributed stochastic neighbor embedding (*t*-SNE), and uniform manifold approximation and projection (UMAP) were consecutively employed for dimensional reduction and visualization. UMAP visualization and cell clustering by Louvain algorithm used the batch-adjusted embeddings from Harmony. FindMarker function using Wilcoxon rank-sum test in Seurat was used to identify the differentially expressed genes (DEGs) in one cluster compared with other clusters (Additional file 2: Table S1). Previously described gene expression patterns were collected and used for cell type annotation [12, 13, 16, 18, 26–28]. The endothelial cell types were processed similarly for further clustering (Additional file 2: Table S2).

#### Identification of key cell types under MCAO condition

Scissor version 2.0.0 was used to identify the key cells highly correlated with the gene module transformation under MCAO condition [29]. Briefly, Scissor exploited the differentially expressed gene modules in bulk RNA-seq data, and subsequently learned the gene module features using Cox regression (with survival data) or logistic regression (with binomial data). Then, the key cells in scRNA-seq data were selected by Pearson correlation according to the similarity of gene expression modules between cells and bulk RNA-seq samples. Our current investigation therefore exploited bulk RNA-seq data from GSE137482 [22] with binomial phenotype data. The filter was set as 0.5 to capture the robust cells contributing to MCAO condition. The Scissor^+^ cells were predictively associated with MCAO condition.

#### Cell scoring and functional annotation

UCell version 1.3 was used to perform rank-based signature enrichment scoring in each cell [30]. DEGs identified in previous analyses were further filtered for statistical significance [false discovery rate (FDR) < 0.01]. The cell-specific signatures were then generated using the top 30 DEGs in each endothelial cell type (Additional file 2: Table S3). Gene set enrichment analysis (GSEA) was used to find the enriched hallmark pathways in a cell-specific or condition-specific manner, based on the DEGs between cell types (e.g., venous versus others) or treatment conditions (e.g., MCAO versus sham), respectively.

#### Trajectory analyses and driving transcriptional factors prediction

Monocle3 version 1.0.0 was used to perform trajectory analyses to predict the differentiation paths between each endothelial cell subtype without prior knowledge of differentiation time or direction [31]. A pseudo-time analysis was thus utilized to describe the asynchronous cellular state. Although the starting point of differentiation is hard to identify without prior knowledge, package CytoTRACE version 0.3.3 can help predict the order of cell differentiation states and stemness of cells [32]. Dorothea version 1.7.0 was further used to identify the transcriptional factors highly activated in each endothelial cell subtype on the basis of the collection of mouse transcription factor (TF) regulons from different types of resources [33]. The regulons with a confidence level of A, B, and C (excluding D and E) were collected for the prediction.

#### Cell communication inference

CellChat version 1.1.3 was utilized to infer the intercellular communication pathways between each cell type [34]. This R tool can quantify the characteristics of cell–cell communication based on the social network analysis, manifold learning, and pattern recognition. To infer the essential communication pathways within endothelial cell types, the data of ECs were extracted from the whole datasets. The cells were also separated as in sham and MCAO conditions to ensure the comparison of cell communications under different conditions.

### In vitro validation of Xbp1

#### Collection of primary BMECs from rats

All studies and procedures were approved by the Animal Care Committee of Xiangya Hospital, Central South University (approval number 201503075). A total of six male Sprague–Dawley (SD) rats aged 3–5 weeks were anesthetized and used to isolate brain microvascular endothelial cells (BMECs). Detailed procedures have been described in previous studies [35]. The culture medium of primary BMECs comprised Dulbecco’s modified Eagle medium (DMEM) and fetal bovine serum (FBS; 10%; GIBCO BRL, Gaithersburg, MD, USA). Primary BMECs were incubated in humidified 5% CO_2_ and 95% air into a 75 cm^2^ flask at 37 °C. Three passages of BMECs were used for further experimentation.

#### Transfection of lentivirus packages into BMECs

To obtain cells stably overexpressing Xbp1 and cells with Xbp1 knockdown, amplification of complete Xbp1 open reading frame (ORF) from previously selected small interfering RNAs (siRNAs) for Xbp1 was conducted using polymerase chain reaction (PCR) [36]. Detailed information on the primers and siRNA sequences is listed in Additional file 2: Table S4. Notably, to acquire restriction sites to construct overexpressed Xbp1 lentivirus, the ORF of Xbp1 was amplified with primers consisting of XhoI and BamHI restriction sites within the 5′ and 3′ termini, respectively. The corresponding vector-Plvx-puro plasmid was also digested with XhoI and BamHI to ensure the formation of sticky ends. Similar protocols were conducted to construct pLVX-shXbp1 plasmid. The preparation of plasmids into lentivirus was implemented as previously mentioned [37]. The measurement of Xbp1 expression level through quantitative real-time PCR (qRT-PCR) was done to determine the efficiency of small hairpin RNA (shRNA) for the following experiments. The grouping based on Xbp1 expression level of BMECs was as follows: sh-Xbp1 (Xbp1 knockdown group), ov-Xbp1 (Xbp1 overexpressed group), and sh-NC (negative-control group, transfected with lentiviral pLVX-shRNA2 plasmid). To achieve infection, the DMEM medium was removed, followed by two washes of BMECs with phosphate-buffered saline (PBS). Then, 0.5 mL of lentiviral suspension with concentration of 1 × 10^8^ IU/mL and multiplicity of infection (MOI) of 30 was added. Polybrene with a concentration of 8 µg/mL was also added to increase the efficiency of infection. Incubation of these cells was conducted at a temperature of 37 °C overnight after infection. After collection, the infected cells were incubated as in our previous study [36]. At 72 h post-incubation, the infected BMECs were passaged in a DMEM medium containing a predetermined dosage of puromycin twice a week. To construct stable cell lines, positively screened cell lines were subcloned by limiting dilution three times, followed by a 1-month culture in a growth medium containing puromycin.

A complete medium was used for the culture of infected BMECs, and the incubation was carried out at 37 °C in humidified 95% air and 5% CO_2_, in a 75-cm^2^ flask. After 24 h and 36 h of culture respectively, gene expression and protein expression were assessed. After 48-h culture, proliferation, apoptosis, tube formation, and migration assay of cells can be performed.

#### Semi-quantitative analysis of RNA

TRIzol reagent (Invitrogen, Carlsbad, CA, USA) was used to extract total RNA from BMECs and PrimeScript RT reagent kit with gDNA Eraser (Takara Bio, Dalian, China) was utilized to perform reverse transcription and obtain cDNA. qRT-PCR was carried out to measure the relative expression level of Xbp1 mRNA. The quantification of qRT-PCR with an ABI PRISM 7500 was achieved with SYBR Green qPCR SuperMix (Invitrogen, Carlsbad, CA, USA). The primers used in the present study are also summarized in Additional file 2: Table S4. The settings of qRT-PCR have been described in our previous research [36]. We used the 2^−ΔΔCt^ method to calculate the fold changes in gene expression. Experiments were done with three repetitions and in duplicate.

#### Western blot

Total protein from BMECs was extracted by lysis buffer (Takara) and ice-cold RIPA, evaluated by the bicinchoninic acid assay (Thermo Scientific, Rockford, IL, USA), and 10% SDS-PAGE was used for separation. Proteins separated were transferred onto nitrocellulose membranes, followed by incubation with the respective primary antibodies (Abcam, Cambridge, MA, USA). Next, a 40-min incubation of nitrocellulose membranes with horseradish-peroxidase-conjugated goat anti-rabbit IgG secondary antibody (1/10,000; Southern Biotech, Birmingham, AL, USA) was carried out. The protein bands were visualized with ECL (Thermo Scientific). GAPDH was utilized as an internal reference gene to normalize the expressions of other proteins.

#### MTS assay

To study the proliferation status of cells in different groups after transfection in 1, 2, and 3 days, the number of cells with high metabolic activity was determined via colorimetric MTS assay (CellTiter 96 AQueous Assay). Briefly, we first washed cells with PBS to clear the complete medium, and then incubated cells with 20% MTS reagent in a serum-free culture medium for 3 h. After that, the cells with high metabolic activity successfully reacted with tetrazolium. After being pipetted into a 96-well plate, the cells were placed into a spectrophotometric plate reader (FLUOstar OPTIMA, BMG Lab Technologies, Germany) to test the absorbance of each well at 490 nm.

#### Annexin V-FITC/PI-stained flow cytometry

Three groups of cells were detached through trypsinization, and rinsed two times with cold PBS. The cells were centrifuged at 3000 rpm for 5 min, then the supernatant was abandoned and the cells were resuspended in 1× binding buffer at a density of 1.0 × 10^6^ cells/ml. For each of the three groups, 100 μL of sample solution was transferred to a Falcon test tube, and each group has four tubes: double-negative tube, 5 μL Annexin V-FITC-positive tube, 5 μL PI-positive tube, and double-positive tube. Then they were incubated in the dark for 15 min at a temperature of 37 °C, after which 400 μL of 1× binding buffer was added to each sample tube. Then the samples were analyzed by FACS (Becton Dickinson), followed by Cell Quest Research Software (Becton Dickinson).

#### Tube formation assay

Tube formation assay was performed according to the manufacturer’s instructions (BD BioCoat Angiogenesis System—Endothelial Cell Tube Formation). Matrigel solution with reduced growth factor was added to an eight-well chamber plate with a volume of 100 μL per well, then solidified at 37 °C. Meanwhile, 2 × 10^5^ BMECs (72 h) were resuspended in 100 µL M199 medium supplemented with 5 μg/mL vascular endothelial growth factors and added to Matrigel containing wells in triplicate and incubated in 37 °C incubators supplemented with 5% CO_2_ for 6 h. Tube formation was observed after 6 h. Tube numbers were observed using 40× and 100× lenses and analyzed by ImageJ software.

#### Transwell migration assay

All cell culture reagents and Transwell chamber were incubated at 37 °C. BMECs were detached by using trypsin and resuspended in M199 (serum-free medium) containing 0.5% FBS. One-hundred microliters of 2 × 10^5^ cells/mL cell suspension was added into the insert of the Transwell (8 µm pore size), while 600 μl of M199 (including 0.5% FBS) was added into the holder of the corresponding Transwell, which was preceded by incubation for 24 h. The cells in the insert could migrate into the holder of the Transwell during this time. Paraformaldehyde (4%) was then utilized to fix the cells for 15 min, and Giemsa solution was used to stain the cells for 15 min. After removal of the cells inside the insert, the migrated cells in the holder of Transwell were observed under a Nikon Eclipse microscope. Nikon Digital Sight system was utilized to image cells. Cells were calculated from 10 views per well × 3 wells using 10× lenses.

### In vivo validation of Xbp1

#### Animal preparation

The current experiments were approved by the Animal Care Committee of Xiangya Hospital, Central South University (approval number 201503075) and were conducted in the Experimental Zoology Department of Central South University. Male adult SD rats weighing 250–280 g were all specific pathogen free (SPF). A total of 36 SD rats were provided by the Laboratory Animal Center of Central South University and used for establishing the middle cerebral artery occlusion (MCAO) model. The rats had free access to food and water under constant temperature (24 ± 2 °C) and controlled light conditions (12 h light/dark cycles). Rats were randomly assigned to three groups (*n* = 12/group): control group (NC group), transfected Xbp1-shRNA group (sh-Xbp1 group), and transfected Xbp1-overexpression group (ov-Xbp1 group). One week prior to modeling, the rats had been injected with virus into the right striatum by micro-injector stereotactically, with NC group injected with lentiviruses encoding a scramble hairpin, sh-Xbp1 group injected with Xbp1-shRNA virus, and ov-Xbp1 group injected with Xbp1-overexpressed virus.

#### Establishment of MCAO rat model

Firstly, the adult rats were anesthetized with pentobarbital sodium (40 mg/kg, i.p.). A modified thread-occlusion method was exploited to establish the focal ischemic stroke rat model. Briefly, we first incised the median anterior cervical to expose the right common carotid artery (CCA), preceded by the exposure of CCA bifurcation. After that, an incision was created and a standard nylon thread was inserted into the common carotid artery, crossing the bifurcation and finally occluding the internal carotid artery (ICA). The depth of insertion was around 18 mm. These rats were killed after 1 day, 3 days, 7 days, and 14 days of MCAO, respectively, and their intact brain was acquired. Subsequently, rat brains were harvested and collected for further experiments.

#### Infarction identification by triphenyltetrazolium chloride (TTC) staining

Fresh rat brains were dissected coronally into 2 mm slices. Then, the slices were incubated in 2% TTC (Sigma-Aldrich, USA) of PBS at 37 °C for 30 min. After that, 4% paraformaldehyde was utilized to fix the brain slices at room temperature for 1 h. The infarct brain tissues cannot be stained by TTC; instead, they can be discriminated via red-dyeing of viable brain tissue. The identification and quantitative analyses of the area of infarction were completed using Image-Pro Plus 6.0 software (Media Cybernetics Inc., Bethesda, MD, USA). The area of infarction was defined as the percentage of white tissue out of total hemispheric volume [38].

#### Hoechst/RECA-1 immunofluorescence staining

Hoechst/RECA-1 was used to visualize the survival of ECs [39]. Analysis was facilitated by the use of a fluorescent nuclear dye, Hoechst 33342 (5 μM). Slides were stained with RECA-1 antibody (rat endothelial cell antigen-I, MA1-81510; Thermo Fisher Scientific Inc.) to visualize endothelial cells. RECA-1 was used at a dilution of 150 and was revealed using donkey anti-mouse IgG (H+L) highly cross-adsorbed secondary antibody, Alexa Fluor™ 594 (Thermo Fisher Scientific Inc., 1:500). For quantification of the area of Hoechst^+^ and RECA-1^+^ endothelial cells, NIH ImageJ software was utilized. The results were displayed as number of positive cells per micrometer.

#### Terminal deoxynucleotidyl transferase-mediated DUTP-biotin nick end labeling (TUNEL)

To detect DNA fragmentation caused by cell death in the ischemic brain, terminal deoxynucleotidyl transferase-mediated dUTP nick end labeling (TUNEL) staining was applied using the In Situ Cell Death Detection Kit (Roche, Mannheim, Germany) for the coronal sections. Labeled sections were counterstained with DAPI, and images were captured under a fluorescence microscope. NIH ImageJ software was utilized to quantify the counts of DAPI^+^ and TUNEL^+^ cells. The results are displayed as the rate of TUNEL^+^ out of DAPI^+^ cells.

#### PCNA/CD31/DAPI immunofluorescence staining

The successful models were anesthetized before removing the nylon thread from the ICA, and then the rats were perfused with 0.9% saline and, subsequently, 4% cold paraformaldehyde. For angiogenesis evaluation, the separated intact brains were fixed with ice-cold 4% paraformaldehyde overnight and then dehydrated in 30% sucrose for 24 h. The coronal brain slices were obtained using a cryostat, and 25-μm-thick slices were used for further experiments. To avoid possible nonspecific binding, brain slices were washed over and then incubated at 4 ℃ on a shaker overnight. After dual-label immunofluorescence staining, an Olympus FV1000 confocal microscope equipped with a four-laser system (Multi AR laser, HeNe G laser, HeNe R laser, and LD405/440 laser diode) was used to obtain images of fluorescence labeling for the ECs’ nucleus. NIH ImageJ software was utilized to quantify the counts of DAPI^+^, PCNA^+^, and CD31^+^ cells. The cells costained with PCNA^+^/CD31^+^ dual labeling were considered to undergo vessel proliferation. The results were displayed as the rate of PCNA^+^/CD31^+^ dual labeling cells out of CD31^+^ cells.

#### Synchrotron radiation phase contrast scanning (SR-PCI)

After anesthesia in rats in the supine position, heparinized saline was rapidly perfused through the heart to avoid blood leftover in the brain, followed by fixation with 10% paraformaldehyde. Rat brains were then harvested and fixed in a 4% paraformaldehyde solution at 4 °C for 24 h. The rinsed brain specimens were dehydrated using ascending gradients of ethanol and were prepared in a fully dehydrated state for Synchrotron radiation phase-contrast scanning. SR-PCI was performed at BL13W1 of the Shanghai Synchrotron Radiation Facility in China, which is a third-generation synchrotron source with an average beam current of 180 mA and 3.5 GeV of storage energy. On the basis of preliminary experimental parameters, the scanning condition has been set at a photon energy of 20 keV, a spatial resolution of 5.2 μm, and a sample-to-detector distance of 50 cm. SR-PCI exploits the principle that the amplitude and phase of X-rays change when interacting with different matters. In our current analyses, the forward diffraction can be defined by the following formula:

$${\text{n}}{\mkern 1mu} = {\mkern 1mu} 1 - \delta {\mkern 1mu} + {\mkern 1mu} {\text{i}}\beta$$

In this formula, “*n*” indicates the complex refractive index of the medium; “*δ*” represents the refractive index decrement that is correlated with the phase shift, and “*β*” represents the linear absorption coefficient. *δ*/*δ* was set to 100 during phase extraction on the basis of preliminary experiments. After the acquisition of the image, the 3D rendered images were reconstructed with Amira (Mercury, Richmond, TX, USA) to visualize the stereological angioarchitecture of the brain. To quantify the parameters of the cerebral vascular network and render the 3D images, we first acquired a distance map by skeletonization, which was achieved by thinning the binary input vasculature for centerline extraction. After that, we analyzed the skeletonized image with the determination of branches and branch points and calculation of vessel parameters including mean vascular diameter, vessel length, bifurcations, and segments [40].

#### Statistical analyses

SPSS 20.0 statistical software (IBM, Armonk, NY, USA) and GraphPad Prism 9.0 was used for data analysis, and measurement data were presented as mean ± standard deviation (SD). The analysis of overall comparison of the between- group measurement indices was performed with one-way analysis of variance (ANOVA), followed by post hoc tests of the least significant difference. Two-way ANOVA was exploited to analyze the contribution of Xbp1 intervention to the phenotype, along with the days after MCAO surgery. Statistical significance was set at *P* < 0.05. Parametric one-way ANOVA was used to compare vascular node and segment of blood vessels between groups for changes in morphological parameters. Tukey’s post hoc test was used to correct for multiple comparisons. Statistical significance of parametric one-way ANOVA and Tukey’s post hoc test was set at* P* < 0.05.

## Results

### Cellular and molecular profiles of mouse brain

To profile brain cells under ischemic stroke condition, we searched the GEO and Sequence Read Archive (SRA) databases. We found two datasets covering the mouse brain scRNA-seq data: GSE167593 and GSE174574. The original scRNA-seq data in GSE167593 were constructed on the basis of the 10X Genomics library and contained 35,968 cells, while after quality control, only 1724 cells were retained (data not shown), suggesting a high proportion of dead cells in this dataset [41]. Since the high-quality cells were too few, this dataset will not be included in our investigation. GSE174574 included scRNA-seq data generated using the 10X Genomics library. This contains the brain cell captured from mice aged 6–8 weeks under post-MCAO (24 h) and sham conditions [20]. After quality control, doublet exclusion, and batch correction, a total of 52,809 cells and 19,276 genes were retained in this study. The cell cycling scores including S phase and G2M phase were also calculated to exclude their possible influence (Additional file 1: Fig. S1A, B).

**Fig. 1:**
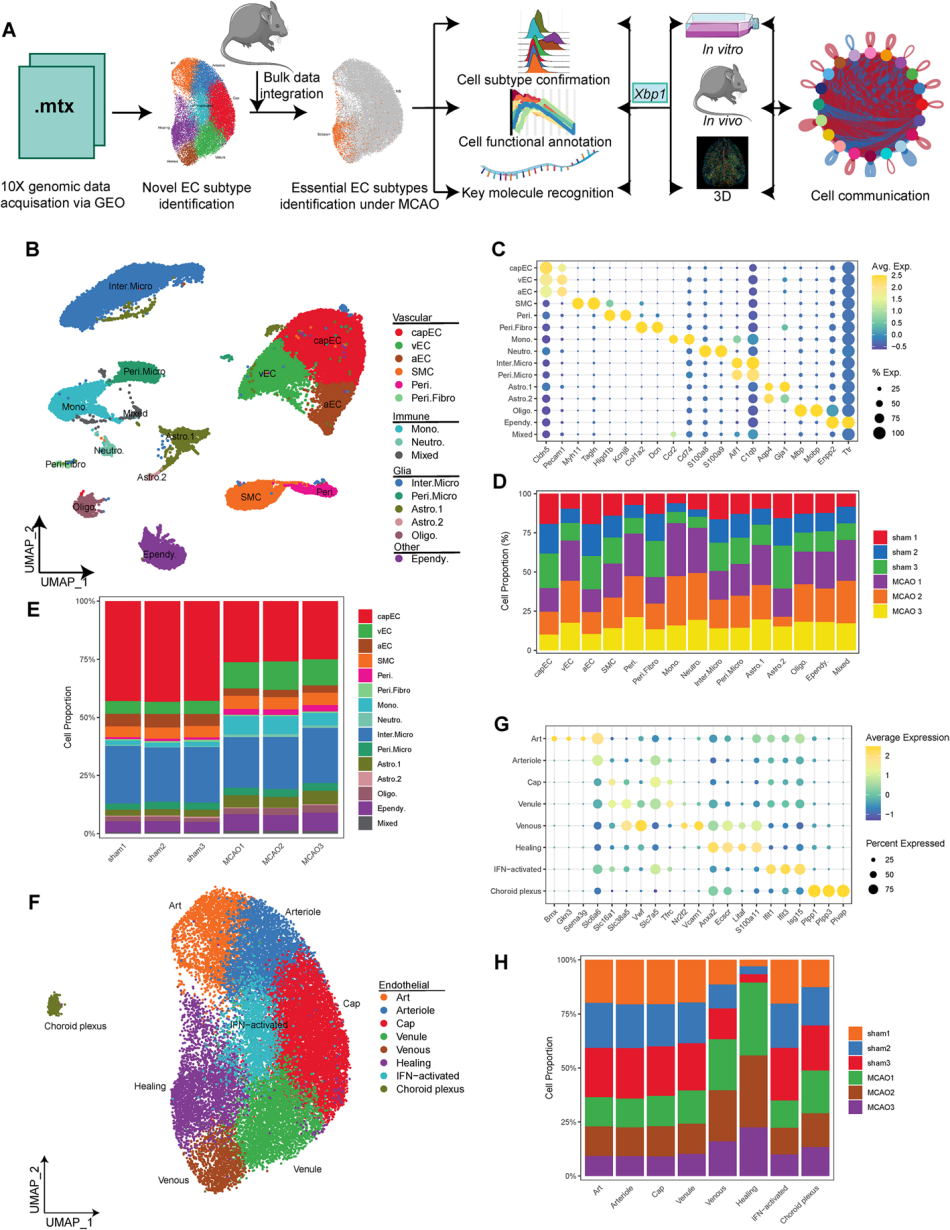
Cellular aberrancy in the mice brain under middle cerebral artery occlusion (MCAO). **A** Quality control, cell identity annotation, endothelial cell (EC) function and driving transcriptional factors identification, development path prediction, and downstream in vitro and in vivo validation were consecutively conducted. **B** UMAP visualization showing different cell types captured from combined sham (*n* = 3) and MCAO (*n* = 3) scRNA-seq data. **C** Dot plot showing the marker gene expression for each cell type. *capEC* capillary endothelial cell, *vEC* venous endothelial cell, *aEC* artery endothelial cell, *SMC* smooth muscle cell, *Peri.* pericyte, *Peri.Fibro* perivascular fibroblast, *Mono.* monocyte, *Neutro.* neutrophil, *Inter.Micro* interstitial microglia, *Peri.Micro* perivascular microglia, *Astro* astrocyte, *Oligo.* oligodendrocyte, *Ependy.* ependyma. **D** Bar plot showing cell proportion in each cell type by sample. **E** Bar plot showing cell proportion in each sample by cell type. **F** UMAP visualization showing EC subtypes in combined sham (*n* = 3) and MCAO (*n* = 3) data. **G** Dot plot showing the marker gene expression for each EC subtype. *Art* artery, *Cap* capillary. **H** Bar plot showing cell proportion in each sample by cell type

On the basis of the previously published gene marker patterns [12, 13, 16, 18, 26–28], we first identified the major brain cell classes in the overall dataset: endothelial cell (*Cldn5*, *Pecam1*), microglia (*C1qb*, *Aif1*), ependyma (*Ttr*, *Enpp2*), smooth muscle cell (*Tagln*, *Myh11*), monocyte (*Ccr2*, *Cd74*), astrocyte (*Aqp4*, *Gja1*), oligodendrocyte (*Mbp*, *Mobp*), pericyte (*Kcnj8*, *Higd1b*), neutrophil (*S100a9*, *S100a8*), and perivascular fibroblast (*Dcn*, *Col1a2*) (Fig. 1B, C and Additional file 2: Table S1). Consistent with the original paper [20], the proportions of most cell types were altered under MCAO condition (Fig. 1D). The proportions of each major cell type in the replicate samples were also similar, confirming the high quality of the generated library (Fig. 1E).

### Identification of healing ECs as a novel endothelial cell subtype

Cerebral vessels were composed of mainly endothelial cells, smooth muscle cells, perivascular cells, and perivascular fibroblasts (Fig. 1B). Previous studies have confirmed that endothelial cells are the main effector cells during angiogenesis [9]. Identified by expression of *Cldn5* and *Pecam1*, endothelial cells were composed of eight subclusters (Fig. 1F). The identities of each endothelial cell subtype were confirmed on the basis of the marker genes related to endothelial arteriovenous zonation: *Bmx*, *Gkn3* (artery), *Nr2f2*, *Slc38a5* (vein), *Slc7a5*, *Mfsd2a* (capillary), *Tfrc*, *Slc16a1* (capillary and vein), *Vwf*, *Vcam1*, and *Slc6a6* (artery and vein) (Fig. 1F and Additional file 2: Table S2). We also identified choroid plexus ECs that were far from other EC subtypes on uniform manifold approximation and projection (UMAP) visualization and highly expressed marker genes *Plpp1*, *Plpp3*, and *Plvap* (Fig. 1F). Among all eight EC subclusters, we noticed one cluster that underexpressed all the marker genes related to endothelial arteriovenous zonation. However, this *Anxa2*^+^EC subtype highly expressed several marker genes that modulated angiogenesis, including *Ecscr*, *Litaf*, *S100a11*, *Igfbp7*, *Col4a1*, etc. (Fig. 1G and Additional file 2: Table S2). We thus named this novel EC subtype “healing EC.” Interestingly, some of these marker genes (*Ecscr*, *Litaf*, *S100a11*) were also expressed in venous ECs, suggesting an intimate relationship between these two EC subclusters (Fig. 1G and Additional file 2: Table S2). The close distance on UMAP visualization further upheld this postulation (Fig. 1F). Nonetheless, the distinction of the two subclusters was still prominent, with healing ECs marked as *Cldn5*^+^ *Pecam1*^+^ *Anxa2*^+^ *Tubb6*^hi^ *Fkbp1a*^hi^, and venous ECs marked as *Cldn5*^+^ *Pecam1*^+^ *Anxa2*^−^ *Tubb6*^low^ *Fkbp1a*^low^ (Fig. 1G, Additional file 1: Fig. S3H and Additional file 2: Table S2). In addition, healing and venous ECs were two major EC subtypes with altered cell number under MCAO condition, suggesting their important roles in MCAO (Fig. 1H).

### Identification of healing ECs as a key endothelial cell subtype under MCAO condition

**Fig. 2:**
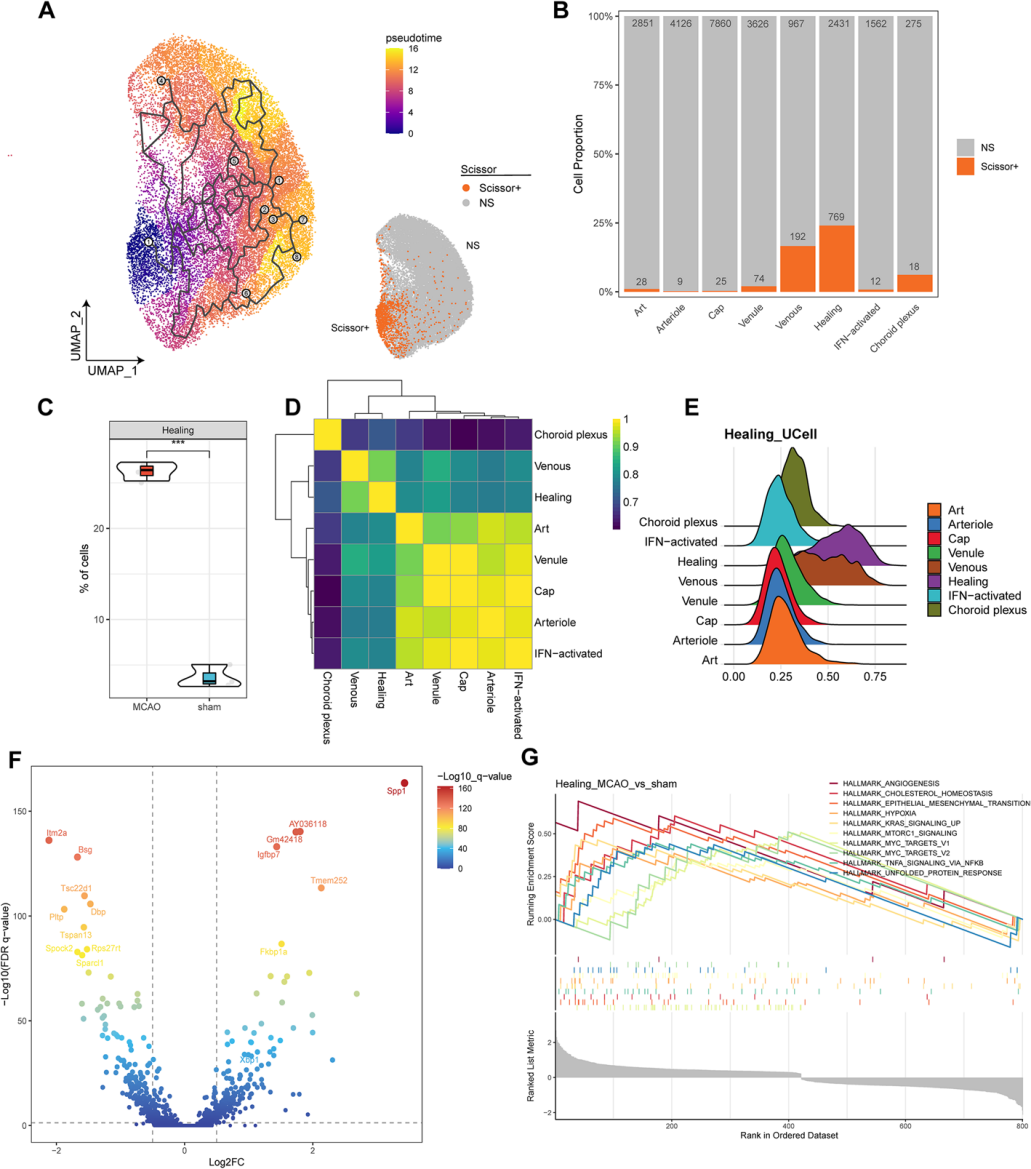
Identification of healing-state endothelial cell as the major EC subtypes changed under MCAO condition. **A** UMAP visualization showing the pseudo-time trajectory of the cells (left) and the Scissor-positive (Scissor^+^) cells under sham (*n* = 3) and MCAO (*n* = 3) conditions (right). Scissor^+^ cells were more relevant to MCAO condition compared with NS cells. **B** Bar plot showing cell proportion in each EC subtype by Scissor results. The number of each cell subtype is labeled. **C** Violin plot comparing the cell proportions of Scissor^+^ healing ECs under sham (*n* = 3) and MCAO (*n* = 3) conditions. Student’s *t*-test was utilized. ***P < 0.001. **D** Heatmap plot showing the overall expression similarity of each EC subtype. **E** Ridge plot showing the UCell signature score of healing-state ECs. The UCell signatures of each EC subtype were defined using the top 30 marker genes filtered for FDR < 0.01. *FDR* false discovery rate. **F** Volcano plot showing the differentially expressed genes in healing ECs between MCAO (*n* = 3) and sham (*n* = 3) conditions. The filter criteria was set as FDR < 0.05 and |log_2_FC|> 0.05. *FC* fold change. **G** Gene set enrichment analysis showing the putative functions of healing ECs under MCAO condition using the filtered differentially expressed genes

It is not sufficient to conclude the importance of a cell subtype exclusively on the basis of its change in number. Instead, its transcriptome can better reflect its relationship with pathological condition. Hence, we exploited a tool named Scissor that utilized the transcriptome of bulk RNA-seq data, to reflect the most relevant cell types under certain conditions. Another bulk transcriptome dataset GSE137482 was thus involved in our present analyses [22]. By learning the transcriptional difference between MCAO (*n* = 12) and control (*n* = 12) samples, the tool successfully targeted a group of Scissor^+^ cells that were predictively essential to MCAO condition in scRNA-seq dataset (Fig. 2A). The Scissor^+^ cells were enriched in cells under MCAO condition and resided mainly in healing and venous ECs, confirming our previous postulation (Fig. 2B and Additional file 1: Fig. S2A). Two-tailed Student’s *t*-test further confirmed the proportional increase of healing and venous ECs under MCAO condition (Fig. 2C and Additional file 1: Fig. S2B). The hidden disadvantage of this method is the mismatch between sample treatment and collection. The brain tissues in GSE137482 were collected from C57BL/6 mice aged 3 or 18 months, which were older than mice in the scRNA-seq dataset. Although this will not greatly affect the differential gene list, it may cause an altered fold change that may influence the prediction [22]. In addition, the tissues were collected 3 days after MCAO in GSE137482 instead of 24 h after MCAO, which may also influence the results. However, the samples from these two datasets were still in the same clinical phase of ischemic stroke, so the results from Scissor remained credible.

To further validate the identity of healing ECs, we compared the expression modules of each EC subtype. Interestingly, healing ECs and venous ECs showed similar expression modules that were different from the other six EC subtypes (Fig. 2D). Consistent with the results, healing and venous ECs also shared a greater number of differentially expressed genes (DEGs) compared with others (Additional file 1: Fig. S2D). Specifically, the DEGs in healing ECs included *Fkbp1a*, *Spp1*, *Xbp1*, etc. (Additional file 1: Fig. S2C). To robustly confirm the independence of healing ECs from other EC subtypes, we used UCell to construct cell-specific signatures based on their top 30 marker gene list (Additional file 2: Table S3, FDR *q*-value < 0.01). The cell scoring of Healing_Ucell indicated a high enrichment in healing ECs, but still, a subset of venous ECs were also enriched (Fig. 2E), meaning at least a proportion of venous ECs were mixed with healing ECs, or in the intermediate state between venous and healing ECs.

Although healing ECs were a disease-related EC subtype under MCAO condition, data also indicated its existence under sham condition (Fig. 1H). We therefore performed differential gene expression analysis on healing ECs between MCAO and sham condition. Several healing-EC-specific marker genes, including *Spp1*, *AY036118*, *Gm42418*, *Igfbp7*, and *Fkbp1a*, were significantly upregulated under MCAO condition (Fig. 2F,  *P*< 0.05, and Additional file 2: Table S5), indicating an enhancement of the healing phenotype after the occurrence of MCAO. Gene set enrichment analysis of the 50 hallmark pathways also indicated an enrichment of essential pro-angiogenic pathways, hypoxia-induced pathways, damage-related pathways, and immune-related pathways in healing ECs under MCAO condition (Fig. 2G,  *P*< 0.05, and Additional file 2: Table S6). Similar results were also seen in venous ECs, further indicating their intimate relationship (Additional file 1: Fig. S2E, F, *P* < 0.05, and Additional file 2: Table S7, S8).

### Healing ECs exhibit differentiation potency into other EC subtypes

Since healing and venous ECs were disease-related EC subtypes and modulators of angiogenesis under MCAO condition, we further wanted to know how they modulated angiogenesis and what differentiation paths they would choose. Monocle3 helped us parse the possible differentiation paths of healing ECs. Choroid plexus ECs were excluded from the analysis owing to its unique distribution and functions in the brain. Consistent with our postulation, healing ECs were predictively a node in the EC differentiation map, indicating its steady cellular state during the differentiation process (Fig. 2A). Interestingly, no differentiation node existed in the venous ECs, suggesting that venous ECs were not a steady cellular state in this process (Fig. 2A). In contrast, capillary ECs harbored five nodes (steady cellular states), showing the high heterogeneity of this EC subtype (Fig. 2A).

The next question was which node acted as the start point of differentiation. Although healing ECs were a steady state, it was also possible that healing ECs were differentiated from other EC subtypes and was instead the result of a response to MCAO stimulation. To address this problem, we utilized CytoTRACE to calculate the state and direction of differentiation in EC subtypes. Apparently, the cells in healing and venous ECs were less differentiated and possessed the highest degree of stemness (Additional file 1: Fig. S3A). Surprisingly, venous ECs possessed even more stemness than healing ECs did, despite the close median in the two EC subtypes (Additional file 1: Fig. S3B). We also calculated the genes correlated most with the CytoTRACE scoring. Genes harboring top ten correlation with CytoTRACE score were all highly expressed in the healing and venous ECs, while healing ECs seemed to express higher than venous ECs did (Additional file 1: Fig. S3G, H). On the basis of the above results, we could therefore conclude that healing ECs acted as the differentiation start point, and that healing ECs were the response rather than result of MCAO stimulation. The pseudo-time trajectory plot showed the refined possible differentiation paths from healing ECs into other EC subtypes (Fig. 2A). However, the conclusions still needed further confirmation since no direct evidence of lineage tracing existed.

We were interested in identifying what defined the cell fate of healing ECs. There were two possible mechanisms: (1) intrinsic factors such as transcription factors (TFs) and other key molecules; (2) extrinsic factors from other cells or the brain microenvironment. To test potential mechanism 1, we utilized the mouse databases of regulon from DoRothEA, a tool used to analyze the TF–target interaction on the basis of several sources of supporting evidence. For an authentic prediction of activated TF in healing ECs, regulons with confidence evidence of A, B, and C (excluding D and E) were extracted. The details of confidence levels are available in the original publication [33, 42, 43]. As a result, DoRothEA predicted many TFs that were specifically activated in each EC subtype (Additional file 1: Fig. S3F). It should be noted that the activated TFs were similar among the seven EC subtypes, in which healing and venous ECs, Art and venule ECs, and arteriole and Cap ECs showed similar TF activation modules, respectively (Additional file 1: Fig. S3F). In spite of the similar TF activation module, the activation strength of each TF still varied among the EC subtypes, indicating a diverse TF-motif binding pattern in each EC subtype (Additional file 1: Fig. S3F). This problem needs to be addressed through further investigation, for example with ChIP-seq or ATAC-seq.

### *Xbp1* served as a key molecule modulating angiogenesis in brain in both physiological and pathological conditions

On the basis of the previously defined trajectory analysis, we further identified several pseudo-time-related and EC subtype-specific molecules, including *Xbp1* (Additional file 1: Fig. S3C). X-box binding protein 1 (*Xbp1*) was generally considered as a transcription factor participating in endoplasmic reticulum (ER) stress and modulating unfolded protein response [44–46], which is an essential pathway enriched in healing ECs under MCAO condition (Fig. 2G,  *P*< 0.05). *Xbp1* was also a marker gene of healing ECs, and was upregulated under MCAO condition in both healing and venous ECs (Fig. 2F; Additional file 1: Fig. S2C, E; Additional file 2: Tables S2 and S5–S8). Among all the variable genes across the seven EC subtypes, *Xbp1* belonged to module 22 (Additional file 1: Fig. S3D). UMAP visualization also confirmed its enrichment in healing and venous ECs (Additional file 1: Fig. S3E). Besides, the expression level of *Xbp1* had a significantly positive correlation with CytoTRACE score (Additional file 1: Fig. S3G, Spearman’s rank correlation test *P* < 0.05), indicating its roles in modulating EC stemness. Previous studies also confirmed the roles of Xbp1 in modulating angiogenesis in the conditions of tumor [47], retinal ischemia [48], cardiac ischemia [49], etc. Combing the above evidence, we therefore supposed that *Xbp1* also played an essential role in modulating angiogenesis under MCAO condition, possibly via enhancing the healing EC phenotype and stemness.

**Fig. 3:**
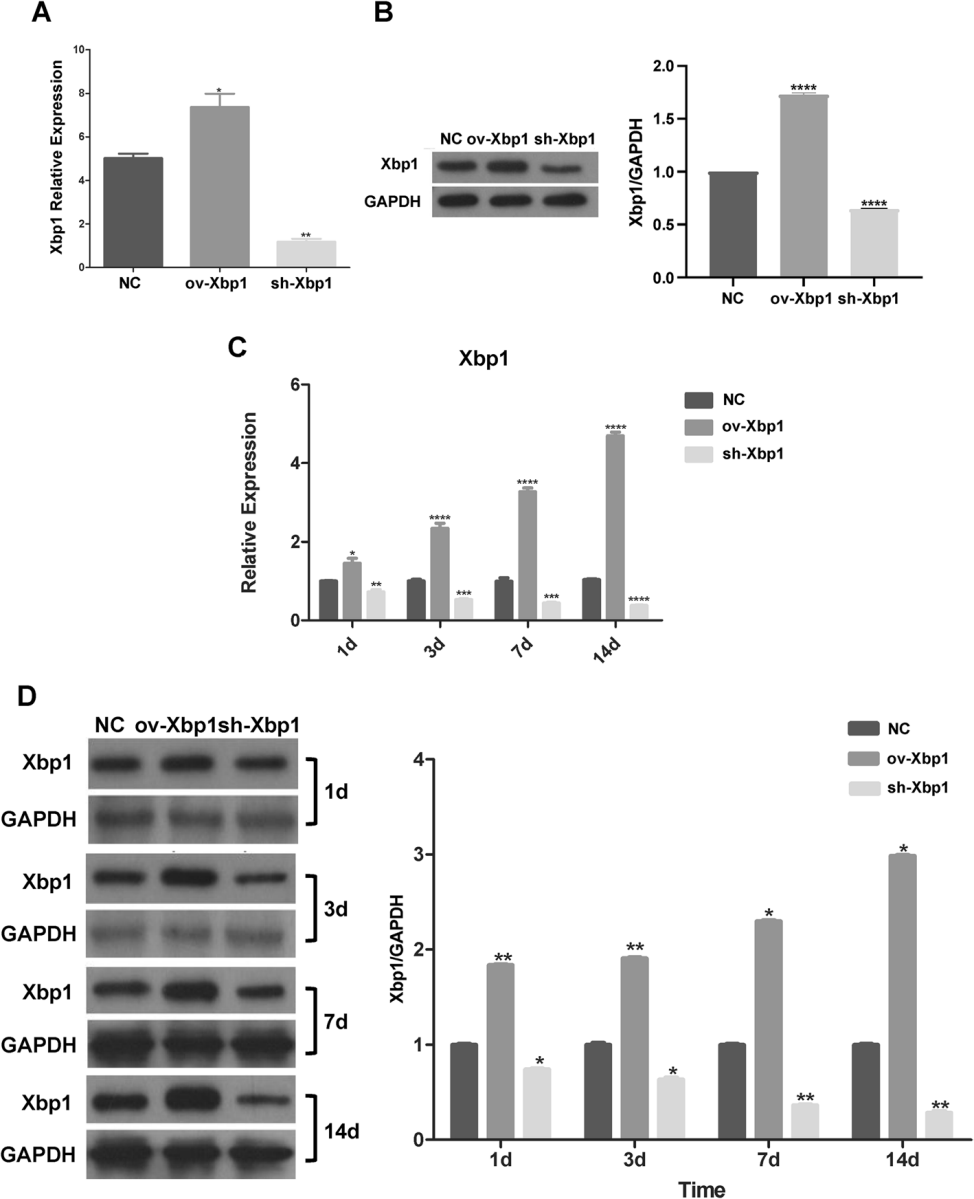
Confirmation of in vitro and in vivo models of Xbp1 overexpression and knockdown. qRT-PCR (**A**) and western blot (**B**) results of the overexpressed Xbp1 and Xbp1-knockdown cells. qRT-PCR (**C**) and western blot (**D**) results of the overexpressed Xbp1 and Xbp1-knockdown rat models. Student’s *t*-tests were conducted. *P < 0.05, **P < 0.01, ***P < 0.001, ****P < 0.0001 compared with the control

**Fig. 4:**
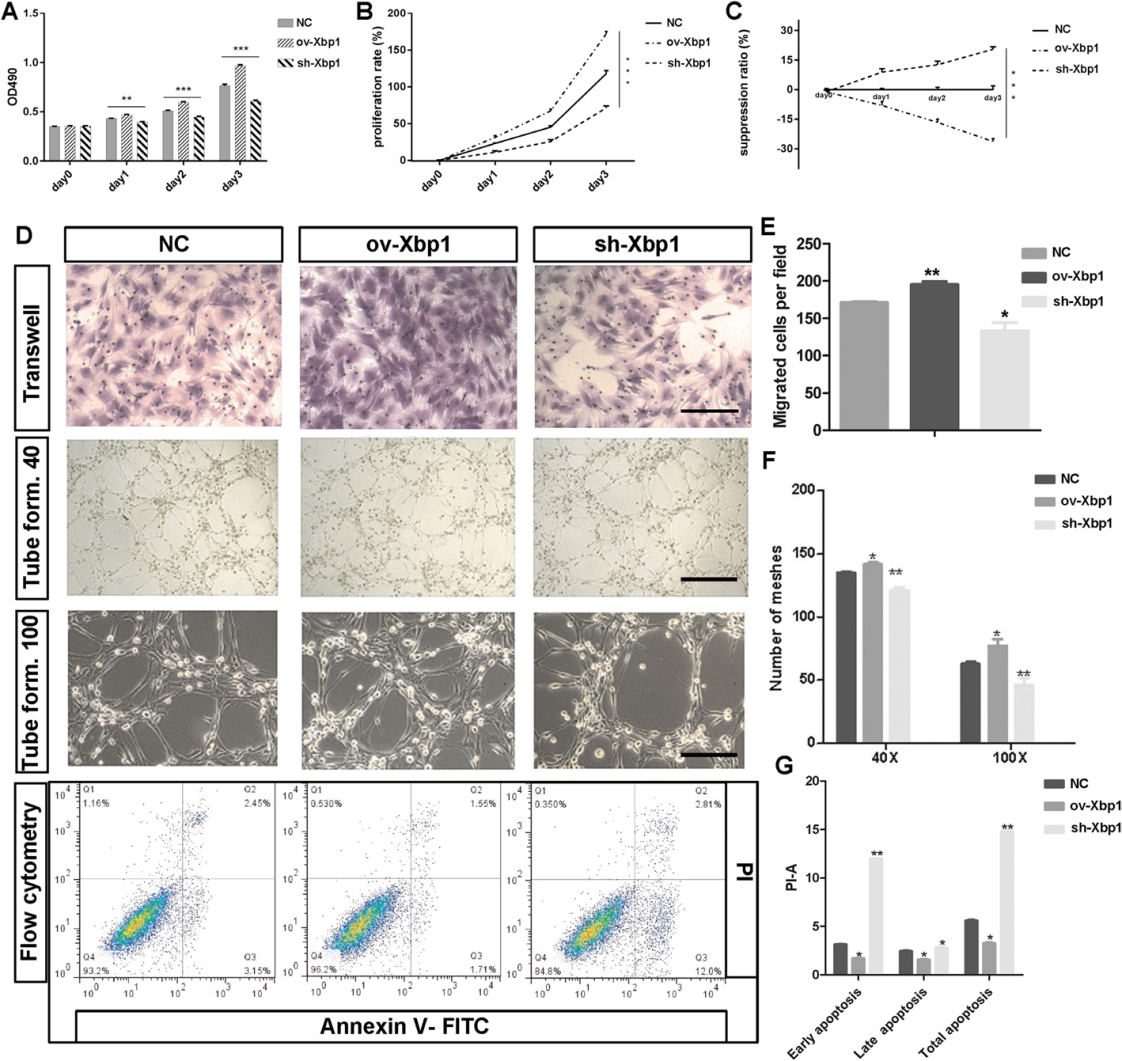
In vitro assays confirming the roles of Xbp1 in improving survival and tube formation, and attenuating apoptosis in BMECs. **A**–**C** MTS assays measured the cell proliferation of brain microvessel endothelial cells (BMECs) after treatment with NC, sh-Xbp1, and ov-Xbp1 for different time intervals, 1 day, 2 days, and 3 days. NC was used as the control group. Proliferation rate = (OD value/(OD value at day 0) − 1) × 100%. Suppression ratio = (1 − experimental OD value/control OD value) × 100%. Results are presented as mean ± SD (*n* = 3 for each groups). One-way ANOVA followed by post hoc test. *P < 0.05, **P < 0.01 compared with the control. **D** Representative figures of Transwell (line 1, scale bar 25 μm), tube formation (line 2, scale bar 50 μm; line 3, scale bar 25 μm), and flow cytometry assays (line 4) under NC, ov-Xbp1, and sh-Xbp1 conditions. **E** Quantitative analysis Transwell assays that showed migration ability of infected BMECs. Results are presented as mean ± SD (*n* = 3 for each groups). One-way ANOVA followed by post hoc test. *P < 0.05 compared to the control. **F** The effect of Xbp1 on the formation of tubes in BMECs was explored with the Matrigel assay at magnifications of 40× (tube formation 40) and 100× (tube formation 100). Results are presented as mean ± SD (*n* = 3 for each groups). One-way ANOVA followed by post hoc test. *P < 0.05, **P < 0.01. **G** Flow cytometry analyzing apoptosis of BMECs treated with sh-Xbp1 and ov-Xbp1 and control groups. Representative diagrams were shown in the quantification of the early and late apoptotic cells. Results are presented as mean ± SD (*n* = 3 in each groups). One-way ANOVA followed by post hoc test. *P < 0.05, **P < 0.01 compared with the control. *BMEC* brain microvessel endothelial cell, *FITC* fluorescein isothiocyanate, *NC* normal control, *OD* optical density, *PI* propidium iodide, *SD* standard deviation, *Xbp1* X-box binding protein 1

To further confirm the roles of *Xbp1*, we conducted a series of in vitro and in vivo experiments. The models of Xbp1 overexpression and knockdown were successfully constructed in both primary BMEC cell line and rodent animals (Fig. 3). On MTS assay, the primary cultured brain microvascular endothelial cells (BMECs) showed a significantly higher optical density (OD) at 490 nm only 1 day after transfection of ov-Xbp1 lentivirus, while a significantly lower OD was also observed in sh-Xbp1 condition, even without pathological stimulation (Fig. 4A,  *P*< 0.01). This effect could exist for at least 3 days (Fig. 4A,  *P*< 0.001). The proliferation and suppression rate of BMECs also varied across different Xbp1 manipulations (Fig. 4B, C,  *P*< 0.001). In addition, upregulation of Xbp1 in BMECs significantly enhanced the migration ability and tube formation ability, while downregulation of Xbp1 showed opposite effects compared with normal control (Fig. 4D–F). BMECs also showed lower apoptosis rate in both early and late phase after upregulation of Xbp1, while opposite effects occurred after downregulation of Xbp1 (Fig. 4D, G). Conclusively, *Xbp1* could promote EC proliferation, migration, and tube formation, and protect ECs from apoptosis in the physiological condition.

**Fig. 5:**
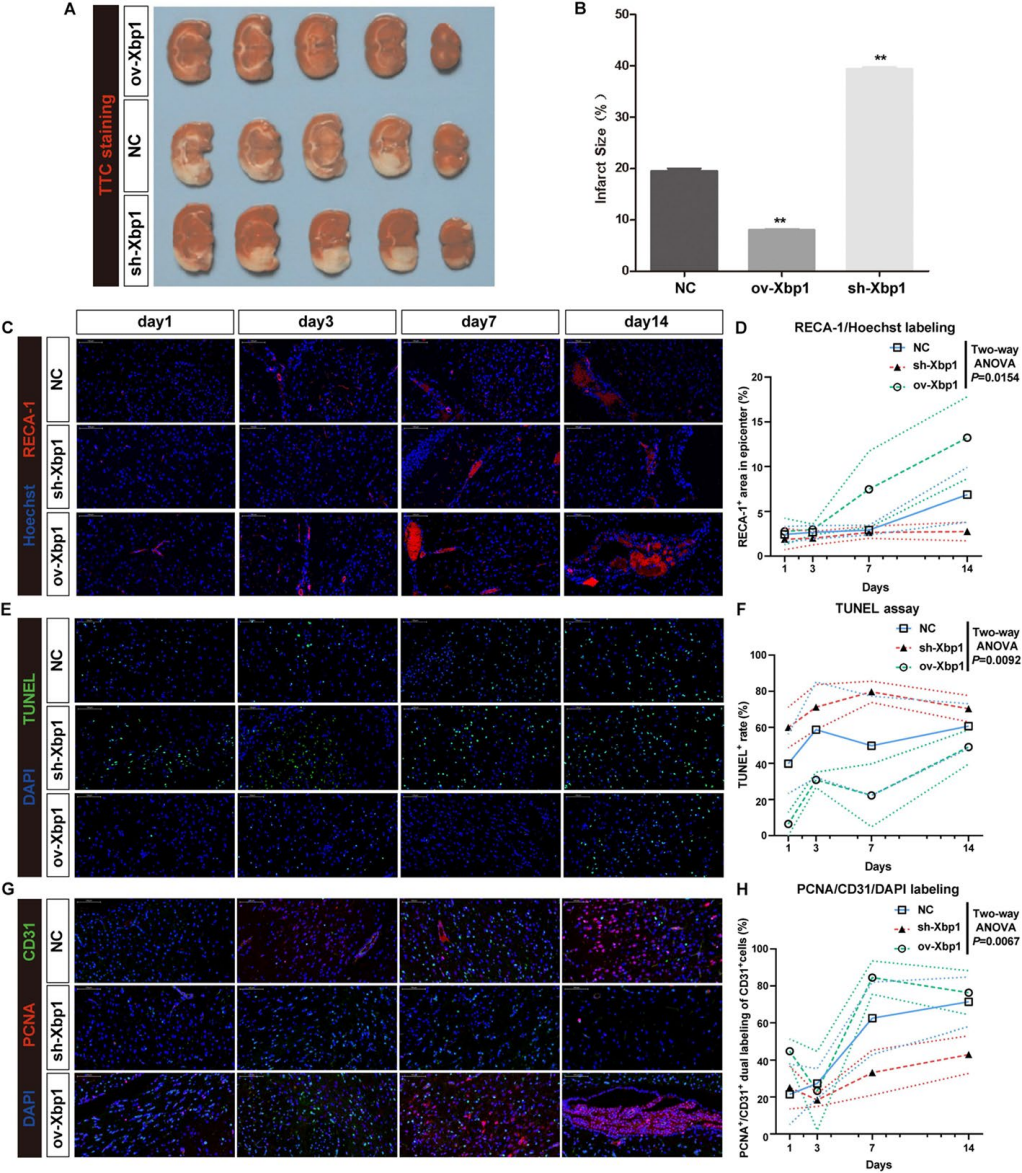
In vivo assays confirming the roles of Xbp1 in improving survival, attenuating apoptosis, and promoting angiogenesis in BMECs. **A** Representative images showing the infarct size of ischemic brain dyed with TTC staining. Regions without staining were considered infarct regions. *TTC* 2,3,5-triphenyl tetrazolium chloride. **B** Bar plot summarizing the infarct size of ischemic brain under different Xbp1 interventions. Results are presented as mean ± SD (*n* = 3 in each group). One-way ANOVA followed by post hoc test. *P < 0.05, **P < 0.01 compared to the control. **C** Representative images showing the survival of endothelial cells by RECA-1 (red) /Hoechst (blue) dual labeling of the brain slices at days 1, 3, 7, and 14 under ov-Xbp1, sh-Xbp1, and NC conditions. Scale bar, 100 μm. **D** Statistics showing the percentage of RECA-1^+^ cell area out of each visual fields at days 1, 3, 7, and 14 under ov-Xbp1, sh-Xbp1, and NC conditions. Results are presented as mean ± SD (*n* = 3 in each groups). Two-way ANOVA. *P* < 0.05 was considered statistically significant. *RECA-1* rat endothelial cell antigen-1, *NC* normal control. **E** Representative images showing apoptosis in the brain by TUNEL (green)/DAPI (blue) dual labeling of the brain slices at days 1, 3, 7, and 14 under ov-Xbp1, sh-Xbp1, and NC conditions. Scale bar, 100 μm. **F** Statistics showing the percentage of the number of TUNEL^+^ cells out of DAPI^+^ cells at days 1, 3, 7, and 14 under ov-Xbp1, sh-Xbp1, and NC conditions. Results are presented as mean ± SD (*n* = 3 in each group). Two-way ANOVA. *P* < 0.05 was considered statistically significant. *TUNEL* terminal deoxynucleotidyl transferase (TdT) dUTP nick-end labeling, *DAPI* 4′,6-diamidino-2-phenylindole. **G** Representative images showing the proliferating endothelial cells by CD31 (green)/PCNA (red)/DAPI (blue) tri-labeling of the brain slices at days 1, 3, 7, and 14 under ov-Xbp1, sh-Xbp1, and NC conditions. Scale bar, 100 μm. **H** Statistics showing the percentage of the number of PCNA^+^/CD31^+^ dual labeling cells out of CD31^+^ cells at days 1, 3, 7, and 14 under ov-Xbp1, sh-Xbp1, and NC conditions. Results are presented as mean ± SD (*n* = 3 in each group). Two-way ANOVA. *P* < 0.05 was considered statistically significant. *PCNA* proliferating cell nuclear antigen, *DAPI* 4′,6-diamidino-2-phenylindole

We further performed in vivo experiments to confirm our postulation. After injection of ov-Xbp1 lentivirus, the rat cerebral infarct size significantly reduced under MCAO condition, while suppression of Xbp1 showed the opposite effect compared with normal control (Fig. 5A, B). For detailed assessment of endothelial cells at successive timepoints after MCAO surgery under different Xbp1 intervention conditions, we performed a series of immunofluorescence microscopic assays. We used rat endothelial cell antigen-1 (RECA-1) antibody to label the ECs [39] and calculated the area of ECs under different conditions (Fig. 5C). The area of ECs was considered the proportion of functional cerebral vessels after MCAO surgery. The results showed a significantly higher proportion of functional cerebral vessels after upregulation of Xbp1, followed by NC and sh-Xbp1 group, especially at days 7 and 14 (Fig. 5D, *P*< 0.05). Meanwhile, terminal deoxynucleotidyl transferase-mediated DUTP-biotin nick end labeling (TUNEL) assays also indicated that a lower proportion of apoptosis occurred after upregulation of Xbp1 compared with the NC and sh-Xbp1 groups, at nearly all four timepoints (Fig. 5E, F,  *P*< 0.01). Cells with a high proliferation activity always highly expressed proliferating cell nuclear antigen (PCNA), and thus can discriminate the proliferating CD31^+^ (PECAM-1, platelet endothelial cell adhesion molecule) endothelial cells from the normal ones. The results also showed a significantly higher proportion of PCNA^+^/CD31^+^ dual labeling out of CD31^+^ cells after upregulation of Xbp1 compared with the NC and sh-Xbp1 groups, especially at days 7 and 14 after MCAO surgery (Fig. 5G, H, *P* < 0.01). All the above evidence indicated the roles of Xbp1 in promoting proliferation and reducing apoptosis under MCAO condition to facilitate angiogenesis, mainly in the recovery phase (≥ 7 days).

### Sophisticated portray of brain microvasculature deciphered detailed angiogenesis influenced by Xbp1 under MCAO condition

**Fig. 6:**
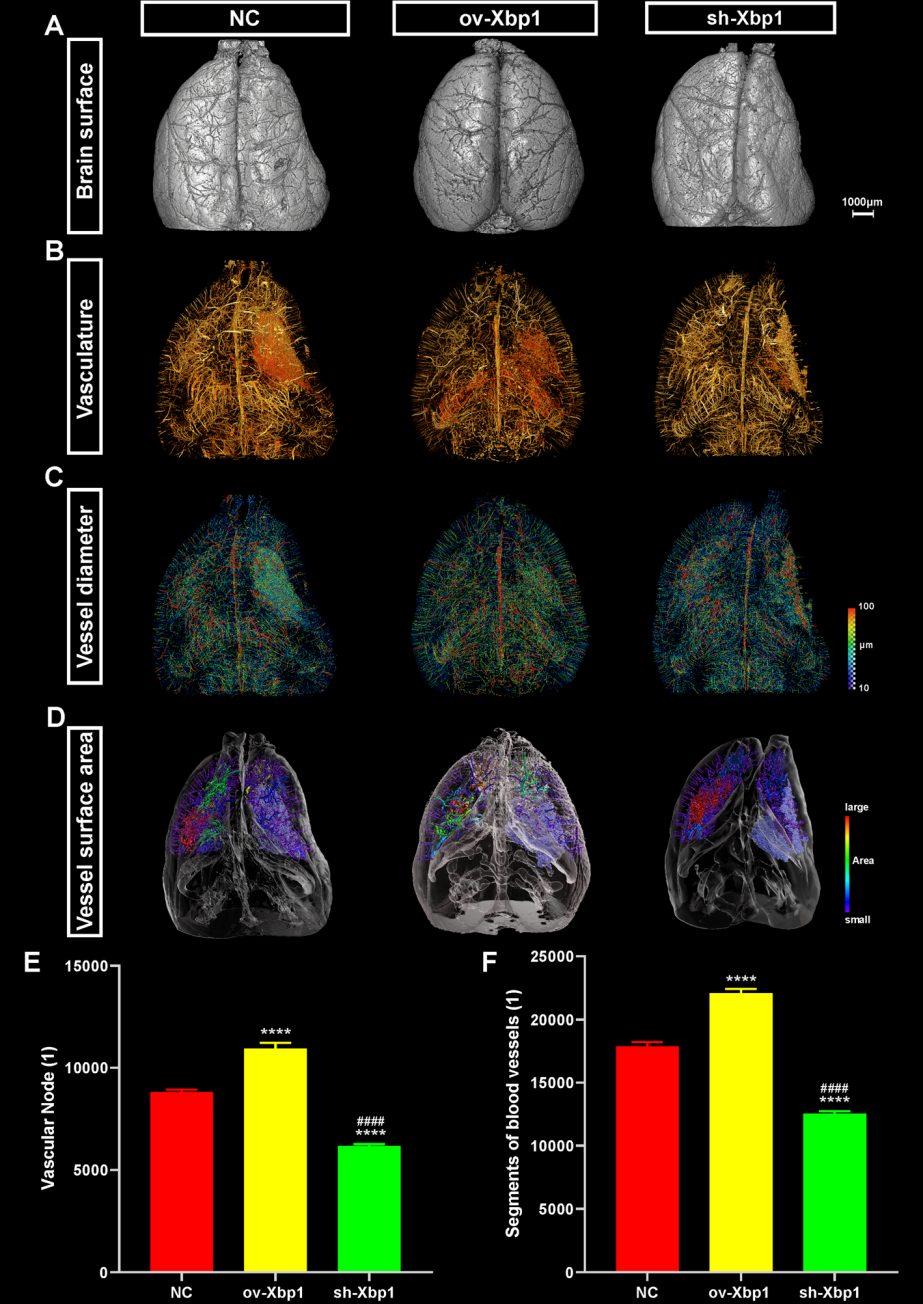
High-resolution 3D tomography showing post-MCAO brain vasculature under different Xbp1 interventions.** A** 3D surface volume images rendering the general appearance and leptomeningeal vasculature of rat brain under NC, ov-Xbp1, and sh-Xbp1 conditions. Scale bar, 1000 μm. **B** 3D vasculature images rendering the path through vessel segment and vascular interconnections under NC, ov-Xbp1, and sh-Xbp1 conditions in the whole-brain context. The red regions indicate the highly connected microvessel network, which were usually seen at the lesion. Scale bar, 1000 μm. **C** 3D vasculature images showing the diameter of the vessels under NC, ov-Xbp1 and sh-Xbp1 conditions in the whole-brain context. The pseudocolor from blue to red indicates the vessels of 10–100 μm in diameter. Scale bar, 1000 μm. **D** 3D images showing the vessel surface area of the regions of interest (ROI) under NC, ov-Xbp1, and sh-Xbp1 conditions. The outline of the brain and important structures is also displayed. The lesion is labeled in light-blue color. Scale bar, 1000 μm. **E** Comparison of the whole-brain vascular nodes in the NC, ov-Xbp1, and sh-Xbp1 groups. Results are presented as mean ± SD (*n* = 3 in each group). One-way ANOVA followed by Tukey’s multiple comparisons test. ****P < 0.0001 compared with the control; ^####^*P* < 0.0001 compared with the ov-Xbp1 group. **F** Comparison of the segments of blood vessels in the NC, ov-Xbp1, and sh-Xbp1 groups. Results are presented as mean ± SD (*n* = 3 in each group). One-way ANOVA followed by Tukey’s multiple comparisons test. ****P < 0.0001 compared with the control; ^####^*P* < 0.0001 compared with the ov-Xbp1 group

Our aforementioned results partly confirmed the functions of *Xbp1* in promoting angiogenesis by immunostaining the surviving and proliferating ECs in two-dimensional (2D) brain slices. However, the robustness of the results was still not satisfying considering the spatial heterogeneity of ischemic injury. The 2D brain slices could not guarantee the representativeness of the whole-brain lesion. To address this issue, we utilized a novel technology called SR-PCI that could capture the whole-brain vasculature at a high spatial resolution while enabling detailed analyses at the regional scale [21]. The MCAO surgeries were conducted on the same one side, to ensure self-control comparison and avoid influence from individual vasculature differences. After reconstruction, we first inspected the overall appearance of the brain under different Xbp1 interventions. Intuitively, cerebral atrophy was the mildest in the lesion side of the ov-Xbp1 group, followed by the the NC and sh-Xbp1 groups (Fig. 6A). The leptomeningeal vasculatures were also preserved the best in the ov-Xbp1 group, indicating a superior ability of collateral compensation (Fig. 6A, middle), while the sh-Xbp1 group preserved the worst (Fig. 6A, right). Three-dimensional (3D) skeletonization of cerebral vasculature showed that the whole-brain-scale blood supply largely varied across different Xbp1 intervention conditions. The conformations of vessels greatly impact their trophic functions. Specifically, the ov-Xbp1 group showed the best-preserved blood-supplying microvessels in the lesion-side corpus striatum with the best vascular network conformation (Fig. 6B, middle), while sh-Xbp1 showed the worst (Fig. 6B, right). The vessels supplying cerebral cortex also preserved the best in the ov-Xbp1 group, followed by the NC and sh-Xbp1 groups (Fig. 6B, and Additional file 1: Fig. S4A). Interestingly, the microvessels were also enriched on the normal side in ov-Xbp1 group, indicating better angiogenesis even in the physiological condition (Fig. 6B, middle). The further vascular diameter analysis confirmed the above conclusions. The newly formed microvessels in the lesioned corpus striatum of the ov-Xbp1 group resembled those of the normal side the best (Fig. 6C, middle, and Additional file 1: Fig. S4B, C) with a diameter of ~ 40–50 μm, indicating the best trophic function preservation of this microvasculature. Under the other two conditions, the microvasculatures were either too narrow in the NC group (Fig. 6C, middle) with a diameter of < 30 μm or too few in the sh-Xbp1 group (Fig. 6C, right). The enlarged vessels in the lesioned side of sh-Xbp1 were possibly the obstructed vessels due to ischemic injury (Fig. 6C, right, and Additional file 1: Fig. S4B, C). Similarly, the vascular surface area in the lesioned prefrontal cortex of the ov-Xbp1 group was also larger compared with the other two groups (Fig. 6D and Additional file 1: Fig. S4D), indicating a better trophic supply in this encephalic region. Statistical analyses of the whole-brain vasculature further confirmed the above conclusions, while the number of vascular nodes and segments were significantly higher in the ov-Xbp1 group and significantly lower in the sh-Xbp1 group (Fig. 6E, F,  *P*< 0.0001). Collectively, by meticulous investigation of the whole-brain and regional micro-vasculature, we confirmed that upregulation of Xbp1 significantly enhanced the angiogenesis and recovered the normal microvasculature at the lesioned site under MCAO condition, especially in the corpus striatum and prefrontal cortex.

### Altered cell communication of endothelial cells under MCAO condition

The above-mentioned analyses in part explain the intrinsic drivers of healing EC phenotype and angiogenesis function. Next, we would like to explore the extrinsic pathways influencing EC subtypes. We exploited a widely used tool, CellChat, which utilizes manually curated database based on Kyoto Encyclopedia of Genes and Genomes (KEGG) and primary literature to infer cell communications [34]. To ensure the comprehensive analysis of cell communications, we integrated the data of EC subtypes and other cells. Compared with the sham condition, the number of interactions between cells generally increased while the interaction strength was impaired (Additional file 1: Figs. S5A S6A, B). The overall statistics confirmed this phenomenon (Additional file 1: Fig. S6C). Specifically, the number of interactions increased mostly owing to the increased receiving pathways in monocytes, suggesting an immune activation process under post-24 h MCAO condition. On the contrary, the EC subtypes were more likely to be a signal sender under post-24 h MCAO condition, including healing and venous ECs (Additional file 1: Figs. S5A and S6A, B). To understand the activated communication pathways in detail, we compared the relative information flow in the endothelial cells under sham or MCAO conditions. The main activated pathways enriched in endothelial cells included mainly pro-inflammatory pathways (MHC-I, IFN-II, TNF, CCL, CXCL) and angiogenesis-related pathways (SEMA3, ANGPT, OSM, ANGPTL, SPP1) under MCAO condition (Additional file 1: Fig. S5B). Both healing and venous ECs showed enrichment in the ANGPTL pathway as a signaling sender (Additional file 1: Figs. S5C and S6D). By analyzing the detailed roles played by each cells, we found that healing ECs and SMCs were most important cells that influenced and sent ANGPT signaling, and that most EC subtypes (except capillary EC) were major receivers (Additional file 1: Fig. S5D). Consistently, the expression of *Angpt2* was upregulated in healing ECs and SMCs, while the receptor *Tek* was more lowly expressed in capillary ECs (Additional file 1: Fig. S5G). We thereafter performed similar analyses on ANGPTL (Additional file 1: Fig. S5E, H), SEMA3 (Additional file 1: Fig. S5F), and OSM (Additional file 1: Fig. S6E) pathways; all these pathways showed high participation of EC subtypes, especially healing and venous ECs. Collectively, these results indicated that EC subtypes profoundly participated in the cell communication process under post-24 h MCAO condition, mainly by introducing pro-inflammatory signals and pro-angiogenic pathways. Although consistent with our previous results, the exact cell communications remain to be validated by other in vitro and in vivo experiments to exclude possible false-positive results.

## Discussion

Angiogenesis is an essential physiological and pathological process that is widely seen in brain development, tumor progression, post-traumatic repair, etc. [50, 51]. Previous studies have investigated angiogenesis after ischemic stroke [5–7]. Endothelial cells, a prominent cell type in brain vasculature, modulate angiogenesis in two patterns: sprouting and intussusceptive [52]. For sprouting angiogenesis, the endothelial cells sprout and form a tip that further becomes a newly formed vessel, while for intussusceptive angiogenesis, the splitting and extending endothelial cells form a new lumen inside the pre-existing vessels, which finally become a new vessel [11, 53]. Owing to the heterogeneity of ECs, the physiological brain vascular single-cell atlas has been established on the basis of human and mouse samples [13, 16, 18, 27, 28], in which the zonation pattern of ECs was found in the brain [12]. Several cell markers of endothelial cells were thus identified, including artery, capillary, and venous ECs [12, 13, 16, 18, 27]. These previously defined cell markers largely expedite the knowledge in several cerebral diseases, including arteriovenous malformation (AVM) [18] and Alzheimer’s disease [54], at a single-cell scale, which identified a capillary-associated variant of ECs and tip-like ECs, respectively. A recent study further identified venous ECs as the main effector EC subtype promoting angiogenesis in the retinal ischemic mouse model [10]. We thus wondered whether similar mechanisms were involved after brain ischemia. Via reanalyzing data from the ischemic mouse brain (GSE174574), we found a specific subset of EC, that is, healing ECs with markers of *Cldn5*^+^ *Pecam1*^+^ *Anxa2*^+^ *Tubb6*^hi^ *Fkbp1a*^hi^. The main evidence supporting the existence of healing ECs was as follows:

(1) The marker gene module was unique in healing ECs. Specifically, healing ECs did not highly express any zonation-related marker genes, but expressed a series of angiogenesis-related marker genes including *Anxa2* [55, 56], *Ecscr* [57], *Litaf* [58], *S100a11* [56, 59],etc. (Fig. 1G). ANXA2 was found to play a key role in decreasing trans-endothelial permeability and sustaining cerebrovascular integrity after injury [55]. Besides, ANXA2, along with its binding partner S100A11, is required for Ca^2+^-dependent plasma membrane resealing in ECs [56, 59]. Ecscr, a cell surface protein expressed in ECs, could promote migration of angioblasts and increase vascular endothelial growth factor (VEGF) receptor sensitivity during zebrafish embryonic development [57]. LITAF can form a complex with another transcription factor, STAT6B, to mediate VEGF transcription regulation, which is important in angiogenesis [58].

(2) We also did not detect a high expression of *Etv2* (embryonic EC) [60–62], *Dll4* and *Vegfr2* (tip cells) [63, 64] , or *Bst1* (self-renewal EC) [65] in healing ECs, indicating its unique role during brain ischemia (Additional file 2: Table S2).

(3) Despite having an intimate relationship with venous ECs, prediction of Monocle3 showed that healing ECs were more likely to be a stable EC subtype under ischemic condition than venous ECs were (Fig. 2A, Additional file 1: Fig. S3F).

(4) Although the median stemness score was predictively lower than venous ECs, healing ECs generally showed higher expression of top ten stemness-related genes (Additional file 1: Fig. S3G, H).

(5) Healing ECs possessed a higher involvement of cholesterol homeostasis (Fig. 2G) instead of glycolysis and amino acid metabolism, showing a special energy metabolism pattern. It also did not overexpress *Pfkfb3* (stalk cells) [66] and Notch signaling, excluding its role as stalk cells (Additional file 2: Table S2).

On the basis of the above evidence, we could conclude that healing ECs were a novel EC subtype under cerebral ischemia condition, most likely a variant of venous ECs. The functional annotation and cell communication analyses indicated their roles in modulating angiogenesis and post-trauma inflammation (Fig. 2, Additional file 1: Figs. S2, S5 and S6).

We further wanted to identify the regulatory factor contributing to the phenotype of healing ECs. Via spatial–temporal analysis of all the highly variable genes, we identified *Xbp1* as the putative molecule. Previous studies have shown that Xbp1 was involved in VEGF signaling and spliced Xbp1 could regulate EC proliferation and angiogenesis in the retina, skeletal muscle, cardiac, and cerebral vasculature [36, 48, 67]. For example, inhibition of the kinase activity of IRE1α, which is responsible for the unconventional splicing of Xbp1, or downregulation of Xbp1 in human umbilical vein endothelial cells (HUVECs) dramatically reduced the tube formation on angiogenesis assay [68]. In isoproterenol (ISO)-induced cardiac hypertrophy mouse models, myocardial capillary density and the expression of VEGF-A were significantly decreased after sh-Xbp1 treatment [67]. In addition, spliced Xbp1 could upregulate endothelial nitric oxide synthase (eNOS) enzyme and regulate its cellular location, contributing to EC migration and angiogenesis [69]. Intriguingly, Xbp1 can also serve as the downstream target of VEGF signaling. Karali et al. reported that VEGF could activate mTORC1 via phosphorylation of PLCγ, leading to the activation of IRE1α and increased levels of spliced Xbp1 in HUVECs [70]. More strikingly, a previous study constructed Xbp1 global knockout or EC conditional knockout mouse models and revealed that spliced Xbp1 was involved in angiogenesis during embryonic development and retinal vasculogenesis angiogenesis in ischemic muscle tissues [48]. In detail, VEGF induces internalization of KDR and its interaction with IRE1α, leading to the phosphorylation of IRE1α. Spliced Xbp1 then facilitates EC cell proliferation in a PI3K/Akt/GSK3β/β-catenin/E2F2-dependent manner and also promotes EC cell size increase [48]. Several in vitro and in vivo experiments confirmed its roles in promoting angiogenesis, not only under cerebral ischemia condition (Fig. 5), but also possibly under physiological condition (Fig. 4). Notably, from the literature discussed above, it seems that only the spliced form of Xbp1 can promote angiogenesis, which is a highly active transcription factor [44]. Further, several studies investigated the therapeutic potentials of targeting Xbp1 and showed that aptamer-conjugated Xbp1 siRNA nanoparticles or scAAV2 vectors encoding the Xbp1 siRNAs could efficiently impair angiogenesis and suppress tumor growth in breast cancer [71–73].

In vitro experiments in the plate and in vivo experiments on brain slices mostly detected the vessel network in a two-dimensional (2D) scale, which may cause bias due to lack of three-dimensional (3D) interconnection and different slice selection, respectively. To overcome the shortcoming, we introduced an SR-PCI method that ensured high-resolution 3D vasculature imaging at whole-brain and regional scales. The SR X-ray sources can provide high-resolution imaging of thick tissues. Meanwhile, SR-PCI utilizes X-ray phase shift, and thus it is far more sensitive to the refraction of X-rays than conventional X-ray absorption imaging. Therefore, SR-PCI is particularly suitable for the detection of delicate biological microstructures without the need for contrast agents [21, 74]. This advanced imaging technique has been used for the 3D visualization of the neuronal network and microvascular network in mouse spinal cord [75, 76]. Microvessels with a diameter reaching 10 μm can be discriminated using SR-PCI. We successfully detected the overall change of vasculature in the MCAO model under different Xbp1 interference conditions. The 3D rendering of whole-brain and regional vasculature indicated higher angiogenesis activity in the Xbp1-overexpressed group. Furthermore, the microvasculature in the lesioned site recovered the best under MCAO condition in the ov-Xbp1 group, especially in the corpus striatum and prefrontal cortex, suggesting a spatial specificity of Xbp1 in promoting angiogenesis. The phenomenon remains elusive, and may be elucidated by spatial RNA-seq technology in the future.

## Conclusions

Endothelial cells are major effector cells modulating angiogenesis after the occurrence of MCAO. A previously generated mouse cerebral vascular atlas has indicated the heterogeneity of endothelial cells under physiological conditions. Herein, by reanalyzing the previously published MCAO single-cell datasets, we successfully identified a novel disease-related EC subtype named “healing EC.” Healing ECs possessed the pan-EC marker and pro-angiogenic genes but underexpressed all the arteriovenous zonation markers. This EC subtype also possessed a high stemness and a tendency to differentiate into other EC subtypes. Via in vitro and in vivo validation, the predicted molecule *Xbp1* was thus confirmed as a key pro-angiogenic modulator under both physiological and pathological conditions. Meticulous 3D whole-brain vasculature analyses further confirmed the roles of *Xbp1* in promoting angiogenesis and recovering normal vasculature conformation. Cell communication analyses further determined the important roles of ECs in cell communication under MCAO conditions, especially with healing and venous ECs as message senders of pro-inflammatory and pro-angiogenic signals.

## Supplementary Information

**Additional file 1: Fig. S1.** Quality control of single-cell RNA sequencing data. **Fig. S2.** Identification of venous endothelial cell as another EC subtypes significantly changed under MCAO condition. **Fig. S3.** The differentiation path and stemness of Healing-state endothelial cell. **Fig. S4.** Demonstration of 3D tomography from different planes. **Fig. S5.** Cellular communication analysis indicating the key roles of endothelial cells in the brain microenvironment. **Fig. S6.** Aberrantly changed cell communication under MCAO condition. (DOCX)

**Additional file 2: Table S1.** Marker genes for all subclusters in the overall dataset. **Table S2.** Marker genes for subclusters in the endothelial cells. **Table S3.** Marker genes for UCell scoring in each EC subtypes. **Table S4.** The primers and shRNA sequences used in PCR. **Table S5.** Differentially expressed gene list of Healing EC between MCAO and sham conditions. **Table S6.** Gene sets enrichment analysis in Healing EC by comparing MCAO and sham condition. **Table S7.** Differentially expressed gene list of Venous EC between MCAO and sham conditions. **Table S8.** Gene sets enrichment analysis in Venous EC by comparing MCAO and sham condition (XLSX)

## Data Availability

The data that support the findings of this study are openly available in the GEO repository at https://www.ncbi.nlm.nih.gov/geo/, reference number GSE137482 (https://www.ncbi.nlm.nih.gov/geo/query/acc.cgi?acc=GSE137482), GSE167593 (https://www.ncbi.nlm.nih.gov/geo/query/acc.cgi?acc=GSE167593) and GSE174574 (https://www.ncbi.nlm.nih.gov/geo/query/acc.cgi?acc=GSE174574). All data generated during this study are included in this published article (and its additional files).

## Abbreviations

MCAO: Middle cerebral artery occlusion
ECs: Endothelial cells
MTS: 3-(4,5-Dimethylthiazol-2-yl)-5-(3-carboxymethoxyphenyl)-2-(4-sulfophenyl)-2H-tetrazolium
Xbp1: X-box binding protein 1
SMC: Smooth muscle cell
scRNA-seq: Single-cell RNA sequencing
SR-PCI: Synchrotron radiation-based propagation contrast imaging
GEO: Gene Expression Omnibus
SRA: Sequence Read Archive
UMAP: Uniform manifold approximation and projection
DEGs: Differentially expressed genes
TF: Transcription factor
BMECs: Brain microvascular endothelial cells
OD: Optical density
RECA-1: Rat endothelial cell antigen-1
TUNEL: Terminal deoxynucleotidyl transferase-mediated DUTP-biotin nick end labeling
PCNA: Proliferating cell nuclear antigen
3D: Three-dimensional
