# Supplementary material for: X-box binding protein 1 as a key modulator in “healing endothelial cells”, a novel EC phenotype promoting angiogenesis after MCAO
Source: Cell Mol Biol Lett. 2022 Nov 8;27:97. doi: 10.1186/s11658-022-00399-5 (PMC9644469; doi:10.1186/s11658-022-00399-5)
Supplement: Supplementary file 1 — Additional file 1: Fig. S1. Quality control of single-cell RNA sequencing data. Fig. S2. Identification of venous endothelial cell as another EC subtypes significantly changed under MCAO condition. Fig. S3. The differentiation path and stemness of Healing-state endothelial cell. Fig. S4. Demonstration of 3D tomography from different planes. Fig. S5. Cellular communication analysis indicating the key roles of endothelial cells in the brain microenvironment. Fig. S6. Aberrantly changed cell communication under MCAO condition. (DOCX) [file 11658_2022_399_MOESM1_ESM.docx]

**Supplementary Figures**

**
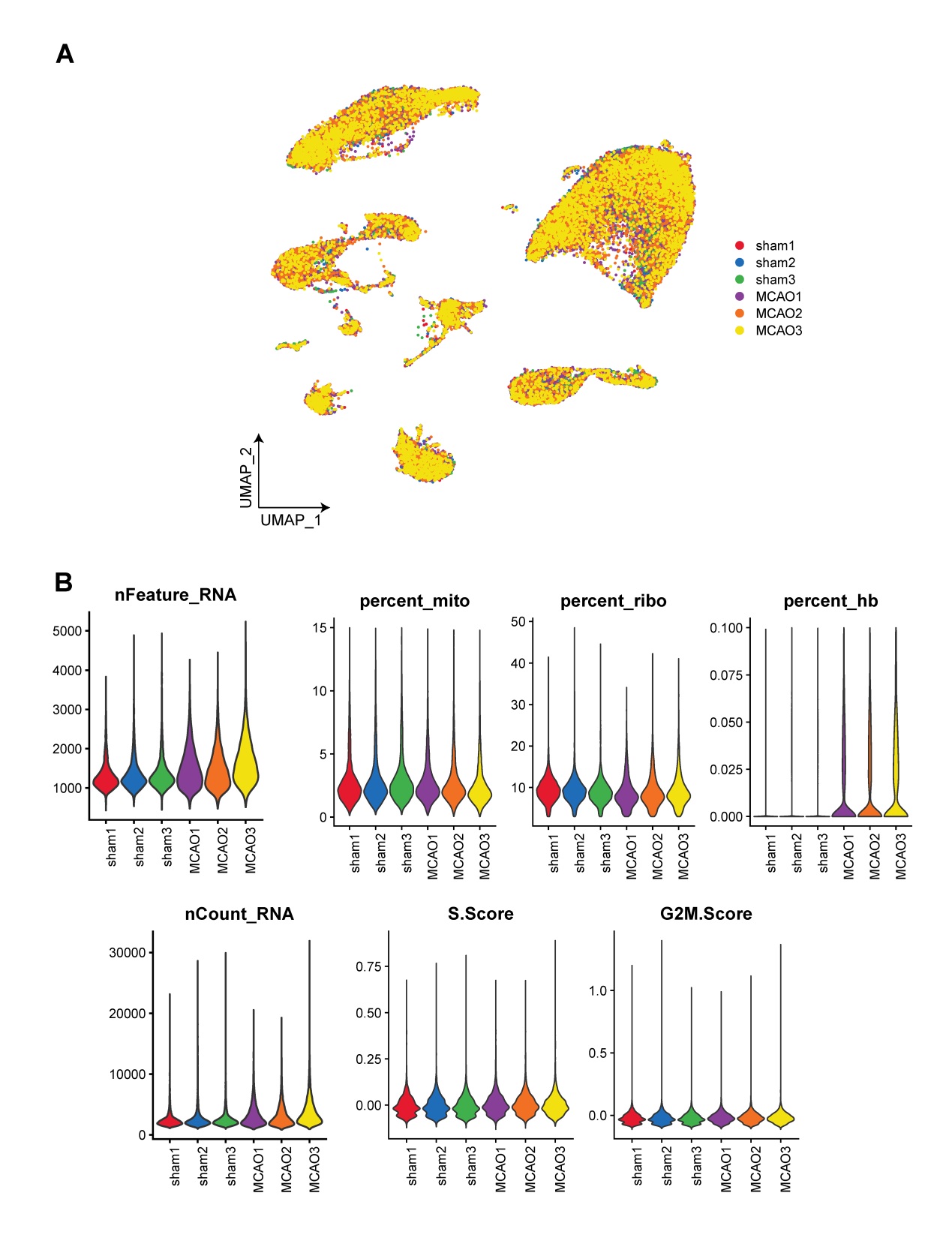
**

**Fig. S1 Quality control of single-cell RNA sequencing data. (A)** UMAP visualization showing the cells grouped by their source samples. **(B)** Violin plots showing the number of detected genes (nFeature), the proportion of mitochondrial (percent_mito), ribosomal (percent_ribo), hemoglobin (percent_hb) genes expression, the mean number of detected molecules (nCount), the scores of cell division state (S and G2M).


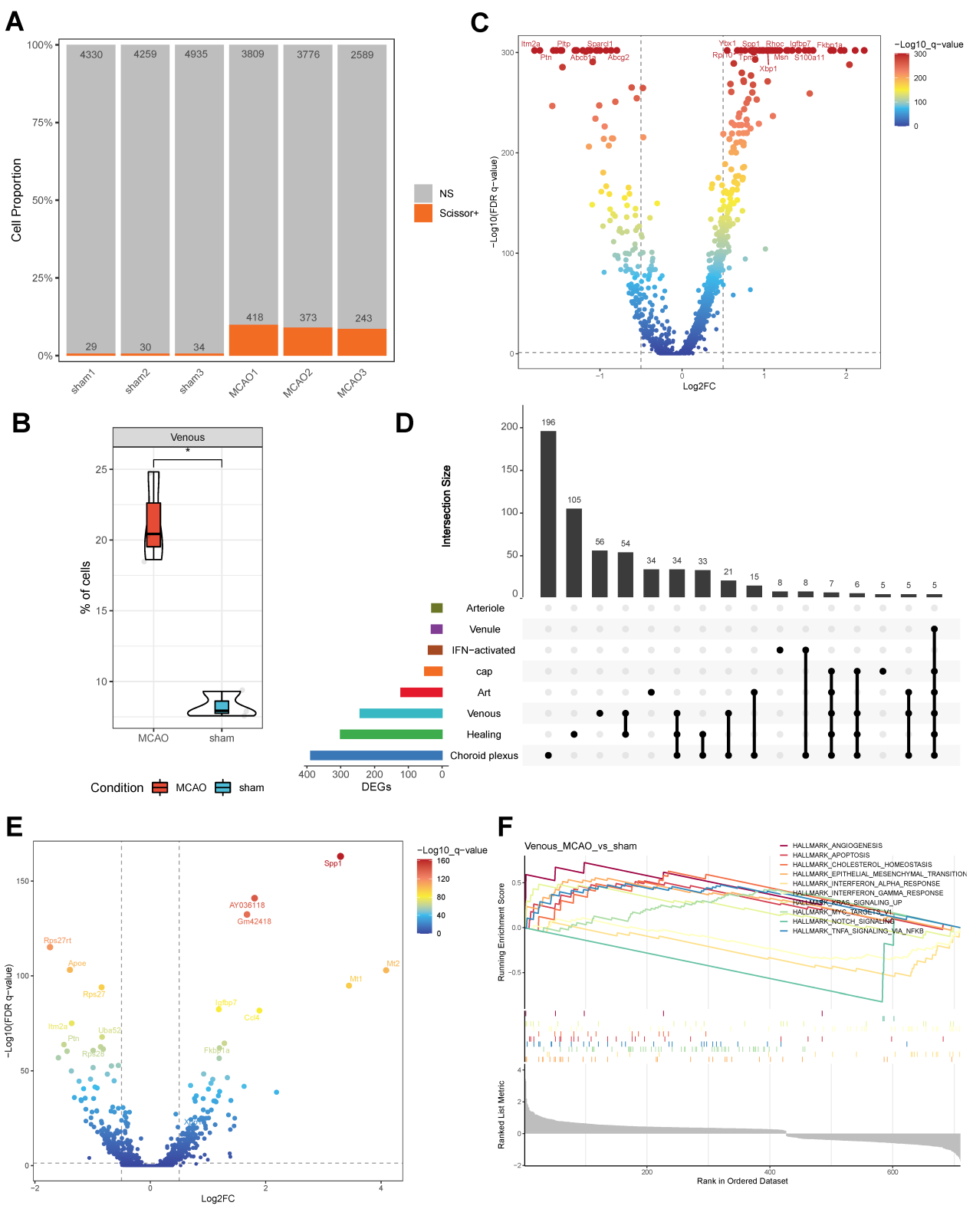


**Fig. S2 Identification of venous endothelial cell as another EC subtypes significantly changed under MCAO condition. (A)** Bar plot showing cell proportion in each samples by Scissor results. The number of each cell subtypes was labeled. **(B)** Violin plot comparing the cell proportions of Scissor+ Venous EC under sham (n=3) and MCAO (n=3) conditions. Student t-test was utilized. **P*<0.05. **(C)** Volcano plot showing the differential expressed marker genes in Healing EC compared to all other EC subtypes. **(D)** Upset plot showing the intersection of DEGs of each EC subtypes. DEG, differential expressed genes. **(E)** Volcano plot showing the differential expressed genes in Venous EC between MCAO (n=3) and sham (n=3) conditions. The filter criteria was set as FDR<0.05 and |log_2_FC|>0.05. FDR, false discovery rate; FC, fold change. **(F)** Gene set enrichment analysis showing the putative functions of Venous EC under MCAO condition using the filtered differential expressed genes.


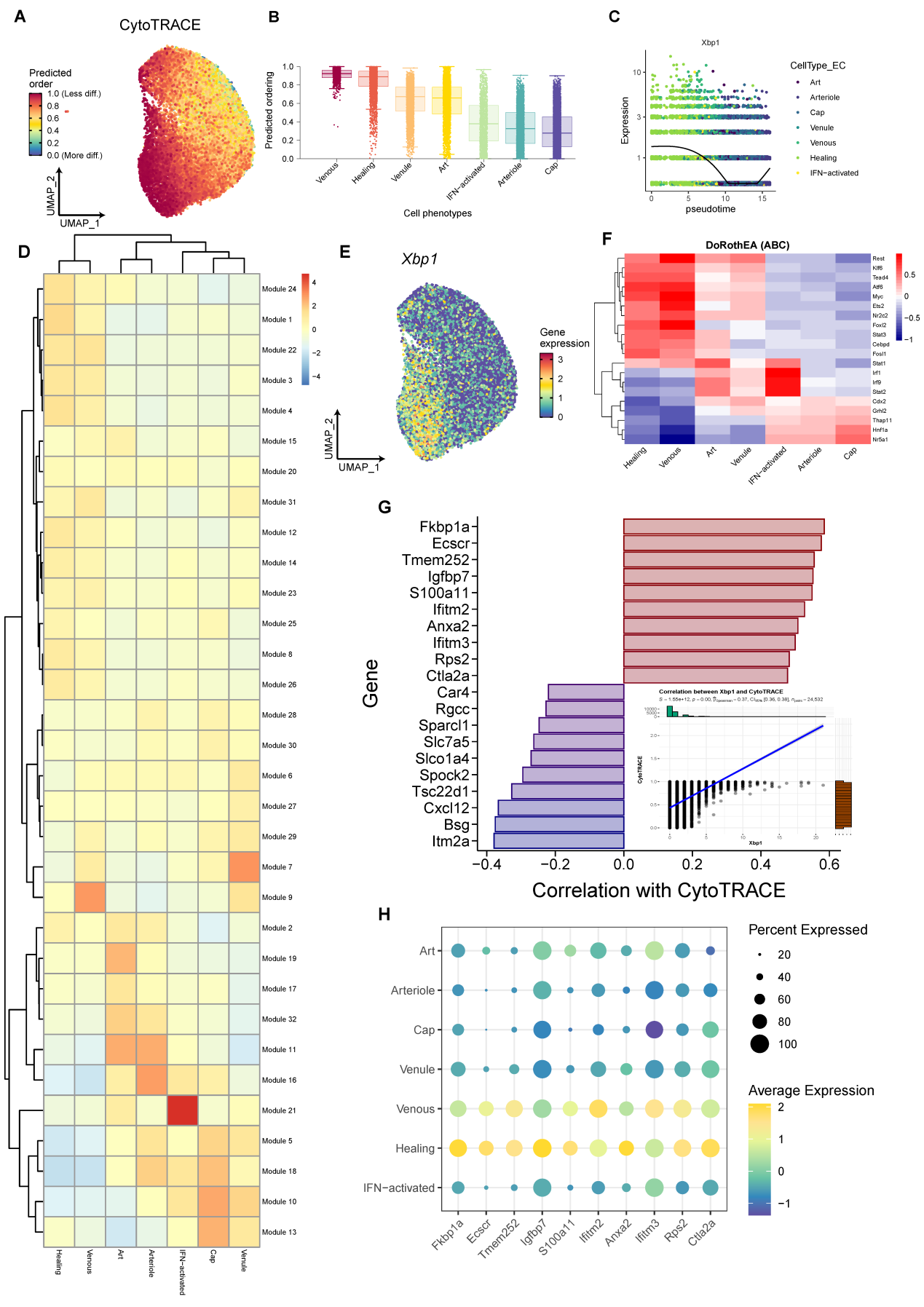


**Fig. S3 The differentiation path and stemness of Healing-state endothelial cell. (A)** UMAP visualization showing the stemness of each cells and the presumptive differential orientation predicted by CytoTRACE. **(B)** Boxplot showing the predicted ordering of differentiation in each EC subgroups. Higher ordering score indicated a lower differentiation state. **(C)** Scatter plot showing the correlation between pseudotime and the expression of *Xbp1.* **(D)** Heatmap showing the 32 aberrantly expressed gene modules in each EC subtypes. *Xbp1* was in Module 22. **(E)** UMAP visualization showing the expression level of *Xbp1* in each cells. **(F)** Heatmap showing the predicted transcription factors of high activities in each EC subtypes. **(G)** Barplot showing the correlation coefficient between the genes and CytoTRACE score to determine the stemness marker genes. Scatter plot showing the correlation between the expression level of *Xbp1* and the CytoTRACE score. **(H)** Dot plot showing the expression level of the 10 stemness marker genes in each EC subtypes.


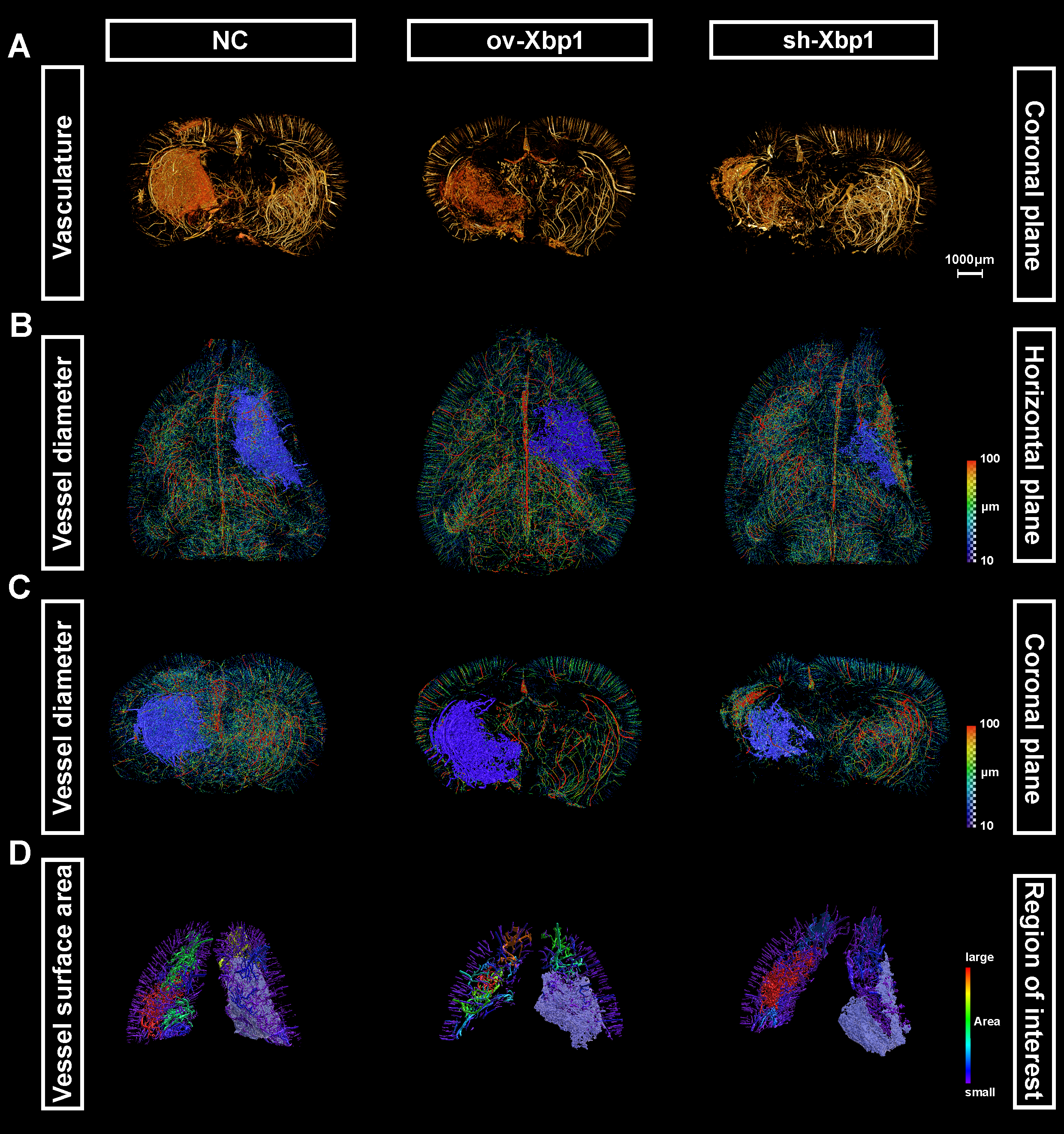


**Fig. S4 Demonstration of 3D tomography from different planes. (A)** Coronal 3D vasculature images rendering vascular skeleton and interconnections under NC, ov-Xbp1 and sh-Xbp1 conditions in the whole brain context. Scale bar=1000μm. **(B)** Horizontal 3D vasculature images showing the diameter of the vessels and the lesion site in the meantime. The pseudocolor from blue to red indicated the vessels of 10 to 100μm in diameter. Scale bar=1000μm. **(C)** Coronal 3D vasculature images showing the diameter of the vessels and the lesion site in the meantime. The pseudocolor from blue to red indicated the vessels of 10 to 100μm in diameter. Scale bar=1000μm. **(D)** 3D images exclusively showing the vessel surface area of the regions of interest (ROI) under NC, ov-Xbp1 and sh-Xbp1 conditions. The lesion was labeled in light-blue color. The pseudocolor from purple to red indicated the relative surface area from small to large. Scale bar=1000μm.


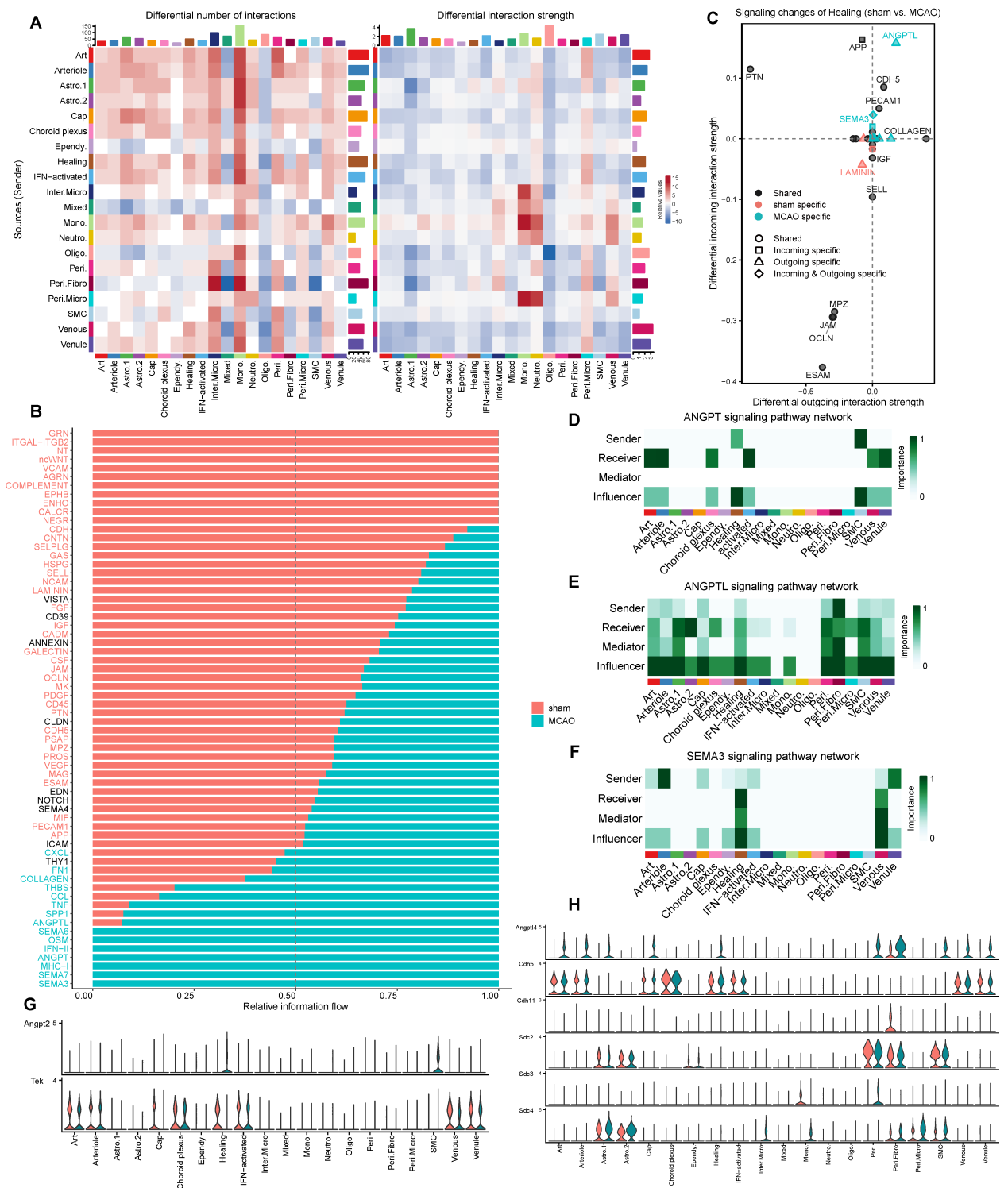


**Fig. S5 Cellular communication analysis indicating the key roles of endothelial cells in the brain microenvironment. (A)** The heatmaps showing the differential number of cell interactions and the differential interaction strength between each cell types. The cells in the rows were sources cells which sent message while the cells in the columns were recipient cells which received the message. **(B)** The barplot comparing the relative information flow in the endothelial cells under sham or MCAO conditions. The cyan names referred to MCAO-specific signaling pathways in the endothelial cells while the red names referred to sham-specific signaling pathways in the endothelial cells. The black names referred to signaling pathways shared by both conditions. **(C)** Scatterplot of dysregulated cell communication pathways in MCAO Healing-state endothelial cells. Red, upregulated in sham; cyan, upregulated in MCAO; black, upregulated in both. Square, incoming pathways; triangle, outgoing pathways; diamond, outgoing and incoming pathways. **(D-F)** Heatmaps showing the roles of each cell types as signaling sender, receiver, mediator or influencer in angiogenesis-related ANGPT, ANGPTL and SEMA3 pathways, respectively. **(G)** Violin plot showing the gene expression level of ANGPT pathway in each cell types under sham (red) and MCAO (cyan) conditions. **(H)** Violin plot showing the gene expression level of ANGPTL pathway in each cell types under sham (red) and MCAO (cyan) conditions.


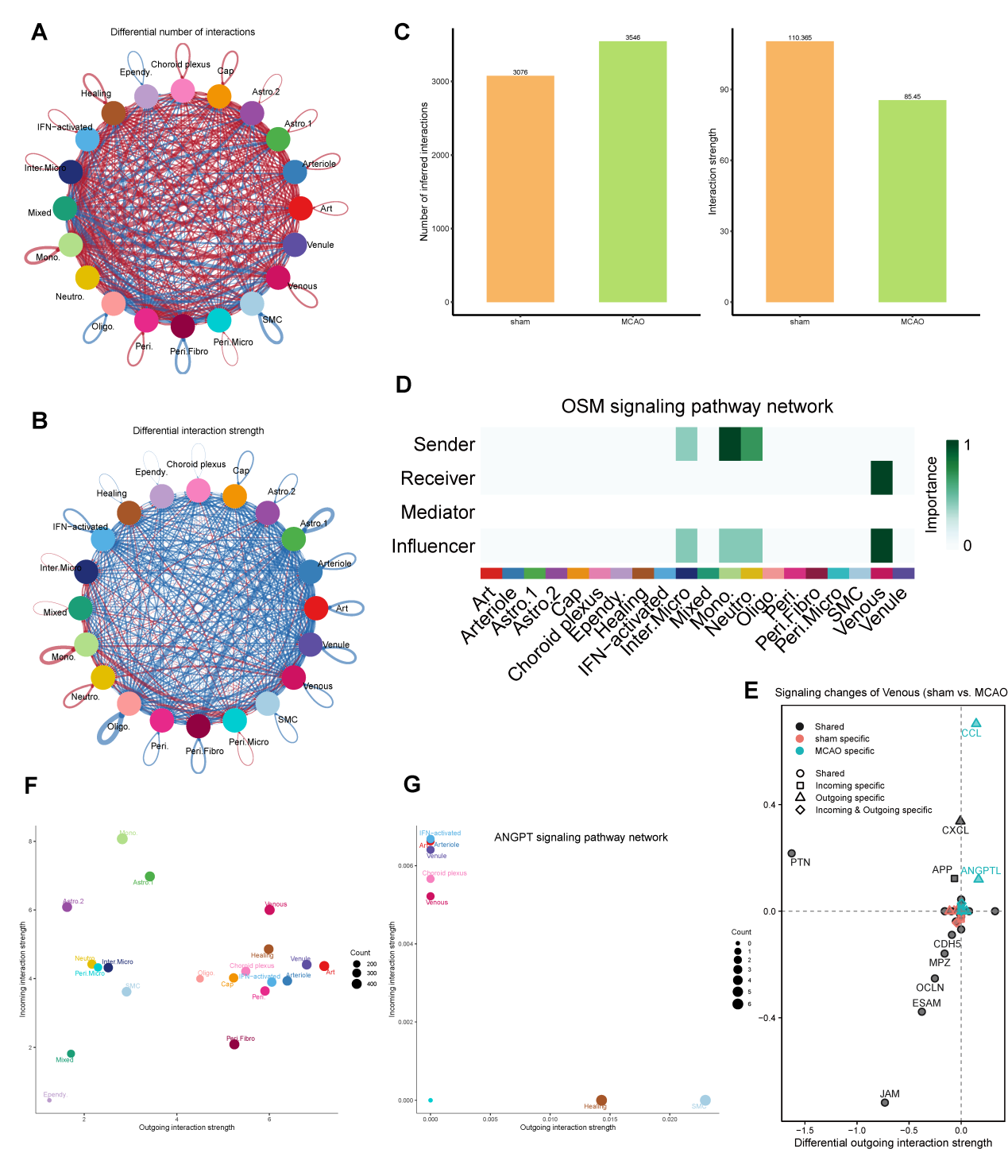


**Fig. S6 Aberrantly changed cell communication under MCAO condition. (A)** Circos plot showing the increased overall interactions of different cell types under MCAO condition. Red lines, increased cell interaction number; blue lines, decreased cell interaction number. The width of cell line referred to the degree of change. **(B)** Circos plot showing the weakened overall interactions of different cell types under MCAO condition. Red lines, enhanced cell interaction strength; blue lines, impaired cell interaction strength. The width of cell line referred to the degree of change. **(C)** Barplot sowing the number of inferred interactions and interaction strength under sham and MCAO condition. **(D)** Heatmaps showing the role of each cell types as signaling sender, receiver, mediator or influencer in angiogenesis-related OSM pathways. **(E)** Scatterplot of dysregulated cell communication pathways in MCAO Venous endothelial cells. Red, upregulated in sham; cyan, upregulated in MCAO; black, upregulated in both. Square, incoming pathways; triangle, outgoing pathways; diamond, outgoing and incoming pathways. **(F)** Scatterplot of outgoing and incoming interaction strength in all cell types. **(G)** Scatterplot of outgoing and incoming interaction strength of ANGPT signaling in endothelial cells.
